# Evaluation of the anti-infective potential of the seed endophytic fungi of *Corchorus olitorius* through metabolomics and molecular docking approach

**DOI:** 10.1186/s12866-023-03092-5

**Published:** 2023-11-18

**Authors:** Arwa Mortada Ahmed, Ayman M. Ibrahim, Ramadan Yahia, Nourhan Hisham Shady, Basma Khalaf Mahmoud, Usama Ramadan Abdelmohsen, Mostafa A. Fouad

**Affiliations:** 1 Department of Pharmacognosy, Faculty of Pharmacy, Daraya University, New Minia City, 61111 Egypt; 2 Department of Pharmaceutical Chemistry, Faculty of Pharmacy, Daraya University, New Minia, 61111 Egypt; 3 Department of Microbiology and Immunology, Faculty of Pharmacy, Daraya University, New Minia City, Minia Egypt; 4 Department of Pharmacognosy, Faculty of Pharmacy, Minia University, Minia, 61519 Egypt

**Keywords:** Antimicrobial activity, *Corchorus olitorius* seeds, Endophytic fungi, Dereplication, Molecular docking.

## Abstract

**Background:**

Endophytic fungi are very rich sources of natural antibacterial and antifungal compounds. The main aim of this study is to isolate the fungal endophytes from the medicinal plant *Corchorus olitorius* seeds (F. *Malvaceae*), followed by antimicrobial screening against various bacterial and fungal strains.

**Results:**

Seven endophytic fungal strains belonging to different three genera were isolated, including *Penicillium*, *Fusarium*, and *Aspergillus*. The seven isolated endophytic strains revealed selective noticeable activity against *Escherichia coli* (ATCC25922) with varied IC_50s_ ranging from 1.19 to 10 µg /mL, in which *Aspergillus* sp. (Ar 6) exhibited the strongest potency against *E. coli* (ATCC 25,922) and *candida albicans* (ATCC 10,231) with IC_50s_ 1.19 and 15 µg /mL, respectively. Therefore, the chemical profiling of *Aspergillus* sp. (Ar 6) crude extract was performed using LC-HR-ESI-MS and led to the dereplication of sixteen compounds of various classes (1–16). *In-silico* analysis of the dereplicated metabolites led to highlighting the compounds responsible for the antimicrobial activity of *Aspergillus* sp. extract. Moreover, molecular docking showed the potential targets of the metabolites; Astellatol (5), Aspergillipeptide A (10), and Emericellamide C (14) against *E. coli* and *C. albicans*.

**Conclusion:**

These results will expand the knowledge of endophytes and provide us with new approaches to face the global antibiotic resistance problem and the future production of undiscovered compounds different from the antibiotics classes.

**Supplementary Information:**

The online version contains supplementary material available at 10.1186/s12866-023-03092-5.

## Introduction

The current health crisis that has arisen from the spread of antimicrobial resistance has become one of the major causes of death occurring worldwide, accounting for approximately more than 700,000 deaths annually [1]. Antibiotic resistance is the inability of antibiotics to inhibit or kill bacteria with their normal dose [2]. The wide use of antibiotics without a precise indication is the main cause of the resistance [3]. Such a condition requires an investigation to discover and develop new classes of antimicrobial compounds [3]. In this sense, Endophytes, a new category of microbial source that can produce a variety of biological components, have major value for study and broad prospects for development [4]. Endophytes are a distinct micro-organisms group; that reside ubiquitously within healthy plants, with important implications for microbial biodiversity, and have beneficial effects on the growth of plants [5, 6]. Like plants, endophytes also produce an array of bioactive secondary metabolites [6]. Endophytic fungi are a significant and hyperdiverse type of endophyte, with an estimated one million distinct fungal taxa [4]. Endophytic fungi are found in all kinds of plants, i.e., trees, grasses, algae, and herbaceous plants, and live in mycelial form in biological association with the living plant [7]. Based on a literary study, endophytic fungi are capable of producing previously undiscovered secondary metabolites with beneficial effects, including antimicrobials, antivirals, antifungals, anticarcinogens, immunosuppressants, and antioxidants [8–12]. Several characteristics of the fungal endophyte interaction still need to be fully elucidated, but fortunately, science is advancing in the search for this understanding [13].

**Fig. 1:**
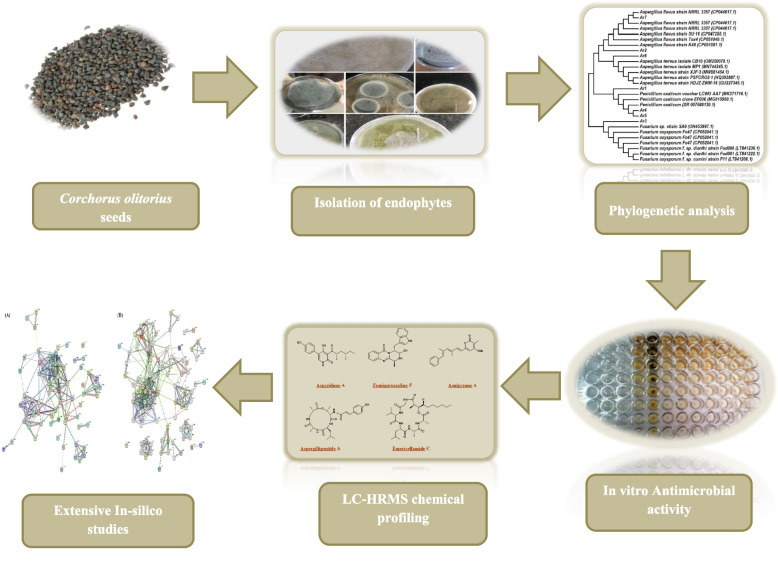
The workflow of this study

*Corchorus olitorius L.* (Family: Malvaceae), commonly known as Jute mallow, is an annual erect herb that grow on roadsides, fields, and home gardens [14]. *C. olitorius* (Jute) is a widely spread native plant of tropical Africa and Asia and has since spread to Australia, South America, and some parts of Europe [15]. Besides having industrial importance in jute production, it also has traditional uses for the treatment of fever, chronic cystitis, aches and pains, dysentery, enteritis, and pectoral pains [15–17]. Consumption of the seeds has been observed to be demulcent, diuretic, purgative, and used in cases of cardiac diseases such as heart failure due to their high content of cardiac glycosides (cardenolides) [16]. *Corchorus olitorius* leaves have been found to have several pharmacological activities, such as anti-inflammatory and antihypertensive effects [18], it also shows hypoglycemic and hypolipidemic effects [19]. *Corchorus olitorius* seeds also show antioxidant and wound healing effects [16], and antiviral activity in the treatment of measles [20]. The aerial parts have antitumor activities against melanoma, leukemia, and osteosarcoma [21]. The plant also shows significant antimicrobial and antifungal activities [22]. *Corchorus olitorius L*., seeds revealed the presence of cardiac glycosides such as coroloside, veticoside, erysimoside, helveticoside, corchoriside A, corchoriside B, strophanthidol, stro-phanthidin, evonoside, chorchorusoside A–E, in addition to three new cardenolide glycosides cannogenol 3-O-β-D-glucopyranosyl-(1→4)-O-β-D-boivinopyranoside, periplo-genin 3-O-β-D-glucopyranosyl-(1→4)-O-β-D-digitoxopyranoside and digitoxigenin 3-O-β-D-glucopyranosyl-(1→6)-O-β-D-glucopyranosyl-(1→4)-O-β-D-digitoxopyranoside [16]. Despite the presence of many pharmacological studies on different aspects of jute, very little is known about the fungal diversity associated with its seeds [23]. This provoked us to explore the endophytic fungi associated with *C. olitorius* seeds that have never been previously investigated, followed by identification of the associated strains by sequencing of the partial 18 S rRNA gene and the internal transcribed spacer (ITS) region. The study was performed on the strains, that belong to a well-recognized genus, and were known to have the ability to synthesize varied secondary metabolites with antimicrobial activity, and were preselected. Additionally, LC-HR-ESI-MS technique of the crude extract was performed to highlight the secondary metabolites, which may share in the antimicrobial potency. Likewise, the molecular docking study predicted the targets that were responsible for the antibacterial and antifungal activities of the dereplicated metabolites to explore the possible mechanisms of their activities. The workflow of this study is depicted in Fig. 1.

## Materials and methods

### Plant material

Fresh seeds of *C. olitorius* were collected from the botanical garden of Botany and Microbiology Department, Faculty of Science, Minia University, Minia, Egypt, in April 2021. The seeds were identified by Prof. Naser Barakat (Botany and Microbiology Department, Faculty of Science, Minia University, Minia, Egypt 28.1220° N, 30.7316° E). A voucher specimen (Corc-3-2021) was deposited at the Department of Pharmacognosy, Faculty of Pharmacy, Minia University, Egypt.

### Chemicals

All chemicals and reagents used in the present study were of high analytical grade, purchased from Sigma Chemical Co. Ltd. (St Louis, MO, USA) and Merck (Germany).

### Isolation and identification of endophytic fungi

According to Strobel et al. protocol, the isolation of the endophytic fungi from *C. olitorius* seeds was performed [24] with slight modifications. Seeds were washed under running tap water, followed by soaking in ethanol for 1 min and then treated with 3% sodium hypochlorite for 3 min, and 30 s wash in 70% ethanol. Seeds were rinsed with autoclaved distilled H_2_O twice and dried on sterile tissue paper. The sterilized seeds were crushed in an autoclaved mortar pestle in 3 mL of sterile water. Sabouraud dextrose agar (Merck) media was used for the isolation of fungal endophytes supplemented with gentamycin and amoxicillin (100 µg / L) to suppress any bacterial contamination. As controls, surface-disinfected, and nondisinfected seeds were also placed on the same agar to check for any contaminated fungi. Serial dilutions were prepared from the grounded plant parts, and 100 µl aliquots from each dilution of 1 × 10^−2^, 1 × 10^−3^, and 1 × 10^−6^, 1 × 10^−9^ were spread on the previously mentioned solid growth media and incubated at 30 °C for 24–72 h. We confirmed that all the epiphytic microbes were removed, as no microbial growth occurred on control agar plates after 30 days of culture. Distinct morphological appearances such as color, shape, and growth pattern of cultured colonies were carefully examined. Distinctly different fungal species were identified and subcultured carefully in fresh petri dishes containing the respective media in which the original colony had appeared until visually free of contaminants. Each different endophyte was assigned a specific code, Ar1, Ar2, Ar3, Ar4, Ar5, Ar6, and Ar7. Long-term storage of isolated strains was achieved in a medium supplemented with 30% glycerol at -80 °C.

### Molecular characterization of the endophytic fungi with 18 S rRNA and phylogenetic analysis

Taxonomic identification of the isolated fungal strain recovered from the *C. olitorius* seeds was achieved by DNA amplification and sequencing of the fungal internal transcribed spacer (ITS) region using the universal primers ITS1 and ITS4 [25]. In brief, DNA was extracted from fungal biomass using the MasterPure Yeast DNA extraction kit (epientre, Madison, Wisconsin). DNA amplification was performed with universal fungal primers NS1 (x) and ITS-4 (x). Sanger sequencing with primer ITS-4 was performed by LGC Genomics (Berlin, Germany). The evolutionary history was inferred with the Maximum Likelihood method based on the Kimura 2-parameter model [26]. The percentage of trees in which the associated taxa clustered together is shown next to the branches. Initial tree(s), for the heuristic search, were obtained automatically by applying Neighbour-Join and BioNJ algorithms to a matrix of pairwise distances estimated using the maximum composite likelihood (MCL) approach, and then selecting the topology with superior log-likelihood value. The tree is drawn to scale, with branch lengths measured in the number of substitutions per site. The analysis involved 32 nucleotide sequences. All positions containing gaps and missing data were eliminated. There was a total of 857 positions in the final dataset. Evolutionary analyses were conducted in MEGA6 [27].

### Fungal fermentation and extract preparation

The seven isolated strains were fermented using the solid-state approach [28, 29]. In the solid treatment, 150 µL of each strain were inoculated and streaked on 20 solid plates of the media: SDA (40 g dextrose, 10 g peptone, and 20 g agar in 1 L distilled water, Merk). After 10 days of growth at 30^0^ C, the agar plates were chopped into small pieces and added to each flask containing ethyl acetate (300 ml) to stop the fermentation. Ethyl acetate is a semi-polar solvent with low toxicity and can attract various metabolites and it also wets and reduces the number of spores that would be carried into the air when opening a cultivation container [30]. The Mycelia was disrupted by using an ultrasonic cleaner (Bransonic®) for 30 min, at 100 W power. The extract was prepared by evaporating the organic phase at a temperature ≤ 40 °C in a rotary evaporator (Heidolph ® 125, 45 °C, 154 rpm).

### LC-HR-ESI-MS metabolomics analysis

LC-HR-ESI-MS metabolic analyses of the fungal ethyl acetate extracts were performed as formerly described by Abdelmohsen et al. [24]. Whereas the gradient elution was applied at 300 µl min^−1^ for 30 min using purified water (A) and acetonitrile (B) with 0.1% formic acid in each mobile phase. The gradient program started with 10% B, increased gradually to 100% B, and continued isocratic for 5 min before linearly decreasing back to 10% B for 1 min on Accela HPLC (Thermo Fisher Scientific, Karlsruhe, Germany) coupled with UV–visible detector and Exactive-Orbitrap mass spectrometer (Thermo Fisher Scientific). High resolution mass spectrometry was carried out utilizing positive and negative ESI ionization modes coupled with a spray voltage at 4.5 kV, capillary temperature at 320 °C, and mass range from m/z 150–1500; so that the highest number of metabolites could be covered. The obtained MS data were processed using the data mining software Mzmine 2.10 (Okinawa Institute of Science and Technology Graduate University, Japan) for deconvolution, peak picking, alignment, deisotoping and molecular formula prediction prior to dereplication. The databases used for the identification of compounds were: METLIN and Dictionary of Natural Products (DNP): http://dnp.chemnetbase.com/faces/chemical/ChemicalSearch.xhtml. Chem Bio Draw Ultra 14.0 software was used for compounds chemical structural drawing.

### Antimicrobial activity screening

Three Gram-negative bacteria (*Escherichia coli* ATCC 25,922, *Pseudomonas aeruginosa* ATCC 27,853, and *Klebsiella quasipneumoniae* ATCC 700,603, together with two Gram-positive bacteria (*Staphylococcus aureus* ATCC 9144 and *MRSA* ATCC 33,591), and one yeast *Candida albicans* ATCC 10,231 were used as test microbes for the evaluation of the antimicrobial activity of the crude extract, in which the experiment was performed in 96 -well flat polystyrene plates according to the method described by Richard A. Ingebrigtsen [31]. The plates were incubated overnight at 37^o^C with 100 µL of test extracts (final concentrations of 500 µg/ml) added to 100 µL of Lysogeny broth (LB broth, Merk), followed by 10 µL of bacterial culture suspension. Ciprofloxacin and fluconazole were used as a positive control. The antibacterial activity of the tested extracts was detected as clearing in the wells after incubation, whereas compounds that had no impact on the bacteria caused the growth media to appear opaque in the wells. By using an ELISA microplate reader, the absorbance was measured after roughly 20 h at OD600.

### *In silico* biological activity predictions

The Way2drug platform provides many computational services for researchers that predict biological activity spectra, cytotoxicity, drug adverse effects, mechanism of action, and interaction with metabolic enzymes for chemical compounds [32]. One of these tools is Way2Drug PASS Online (Prediction of Activity Spectra for Substances) which predicts the biological activity spectra of chemical compounds by analyzing the structure-activity relationship of more than one million molecules approved with biological uses. PASS quantitatively classifies the activity of compounds according to their probability to be biologically active or not, with an average accuracy of prediction approximately equal to 95%. The chemical structure of molecules 1–16 were uploaded to PASS Online as an MOL file separately and the activity results are listed. The compound is likely to show the predicted activity experimentally if 0.7 > Pa > 0.5. Compounds with Pa higher than 0.5 were chosen for further analysis. AntiBac-Pred (http://www.way2drug.com/antibac/ ) and AntiFun-Pred (http://www.way2drug.com/micF/ ) are tools based on PASS Online and used for prediction the range of antimicrobial action of studied compounds on the microbial species and strains [33]. The studied compounds were entered into these tools and evaluated compounds against 353 bacterial strains and 38 fungi. The results are listed as confidence ratio where the higher the values, the higher the confidence of activity against the corresponding microbial species.

### Prediction of the potential protein targets of the annotated compounds

#### Construction of protein-protein Interaction (PPI) network

The potential targets of the annotated compounds were predicted by using the PharmMapper server. PharmMapper is an updated integrated pharmacophore web-server that uses reverse molecular docking for potential target identification [34]. Compounds were uploaded to PharmMapper server as MDL/sdf files. The standard parameters were set as the default values, where ‘Gerenrate conformers’ are allowed with 300 conformations as the maximum. Also, Energy minimization is set as “Yes”and the target set selected for the pharmacophore mapping is set as “Druggable Pharmacophore Models (v2017, 16159)”. For the annotated compounds with predicted antibacterial/antifungal activity, targets with *E. coli/ C. albicans* as the source organism were collected for further analyses.

STRING database 11.5 (https://string-db.org/) aims to collect and integrate the information of all functional interactions between the expressed proteins. The targets obtained from PharmMapper were analyzed and compared using STRING. The targets were imported into STRING, where “*Escherichia coli*” was chosen from optional species, and the medium confidence (0.4) was selected as the required interaction score. The targets protein–protein interaction (PPI) network was obtained and extended enrichment feature was used as necessary.

#### Gene Ontology (GO) and Kyoto Encyclopedia of genes and genomes (KEGG) enrichment analysis

For better understanding the biological functions and pathways of these collected proteins, functional enrichment analysis was performed through Gene Ontology (GO) and Kyoto Encyclopedia of Genes and Genomes (KEGG). GO provides the analysis of gene function based on the biological process (BP), cellular compound (CC), and molecular function (MF). Also, KEGG provides biological pathways associated with genes. GO was performed and visualized using ShinyGO (http://bioinformatics.sdstate.edu/go/) 0.76.3 with FDR < 0.05. While KEGG was visualized using SRplot tool (https://www.bioinformatics.com.cn/en).

#### Identification of potential protein targets

To visualize and analyze the PPI network, the target proteins were exported from STRING to the Cytoscape 3.9.1 software (http://cytoscape.org/), and the PPI network’s targets were ranked using the cytoHubba plugin using 12 different metrics (EPC, MCC, DMNC, MNC, Closeness, Degree, BottleNeck, Betweenness, Stress, Eccentricity, Radiality, and Clustering Coefficient), and the coinciding genes were then identified as the hub genes. Molecular docking was performed between known key targets and the annotated compounds for prediction of their binding interactions.

### Molecular docking

Molecular docking or computer-aided drug design (CADD) is one of the in-silico techniques that are frequently used in drug design, as it provides a simulation of a candidate ligand binding to a receptor, which make it a promising technique in finding out the potent drugs through virtual screening of metabolites databases [35]. Molecular docking was performed using AutoDock. The crystal structures of the proteins were obtained from RCSB Protein Data Bank (http://www.rcsb.org/). The input files for molecular docking were prepared using Discovery Studio (DS) 2016 client and AutoDock tools bundled with MGL tools (version 1.5.7) [36]. Proteins were prepared by the removal of water molecules and small molecular ligands, addition of polar hydrogens, charges. The 3D structure of ligands was retrieved from the PubChem database as a single file in 3D-spatial data file (SDF) format. The structures of ligands were imported into DS 2016 and minimized using a universal force field and saved in PDB format. The gasteiger charges and polar hydrogens were added, and the ligands were set up for the rotatable bond. The prepared protein and ligand files were then converted into PDBQT format, which serves as an input file for AutoDock 1.5.7 for molecular docking. The active site of the ligands was chosen according to a literature survey and selected as the active grid center. The dimensions of grid box were chosen to include all atoms of the ligands. The molecular docking was proceeded and the protein-ligand conformation with the lowest binding energy was chosen and visualized by DS 2016 client.

## Results and discussion

### Isolation, identification, and phylogenetic analysis

Based on the morphological characteristics, 17 fungal isolates with distinct colonies were isolated. Seven isolates were selected according to their cultural characteristic appearance for molecular characterization. From 18 S rRNA gene sequence analysis via the BLASTn tool of the National Center of Biotechnology Information (NCBI), the isolates Ar1, Ar4, and Ar5 belong to the genus *Penicillium*, while the isolates Ar2 and Ar7 belong to be *Aspergillus*, the isolate Ar3 belongs to genus *Fusarium* and the isolate Ar6 were identified to be *Aspergillus* Table 1. The sequences were submitted to the GenBank database with the accession numbers OQ520282, OQ520324, OQ520328, OQ520319, OQ520320, OQ520330 and OQ520325 for the isolates Ar 1, 2, 3, 4, 5, 6, and 7 respectively.

**Fig. 2:**
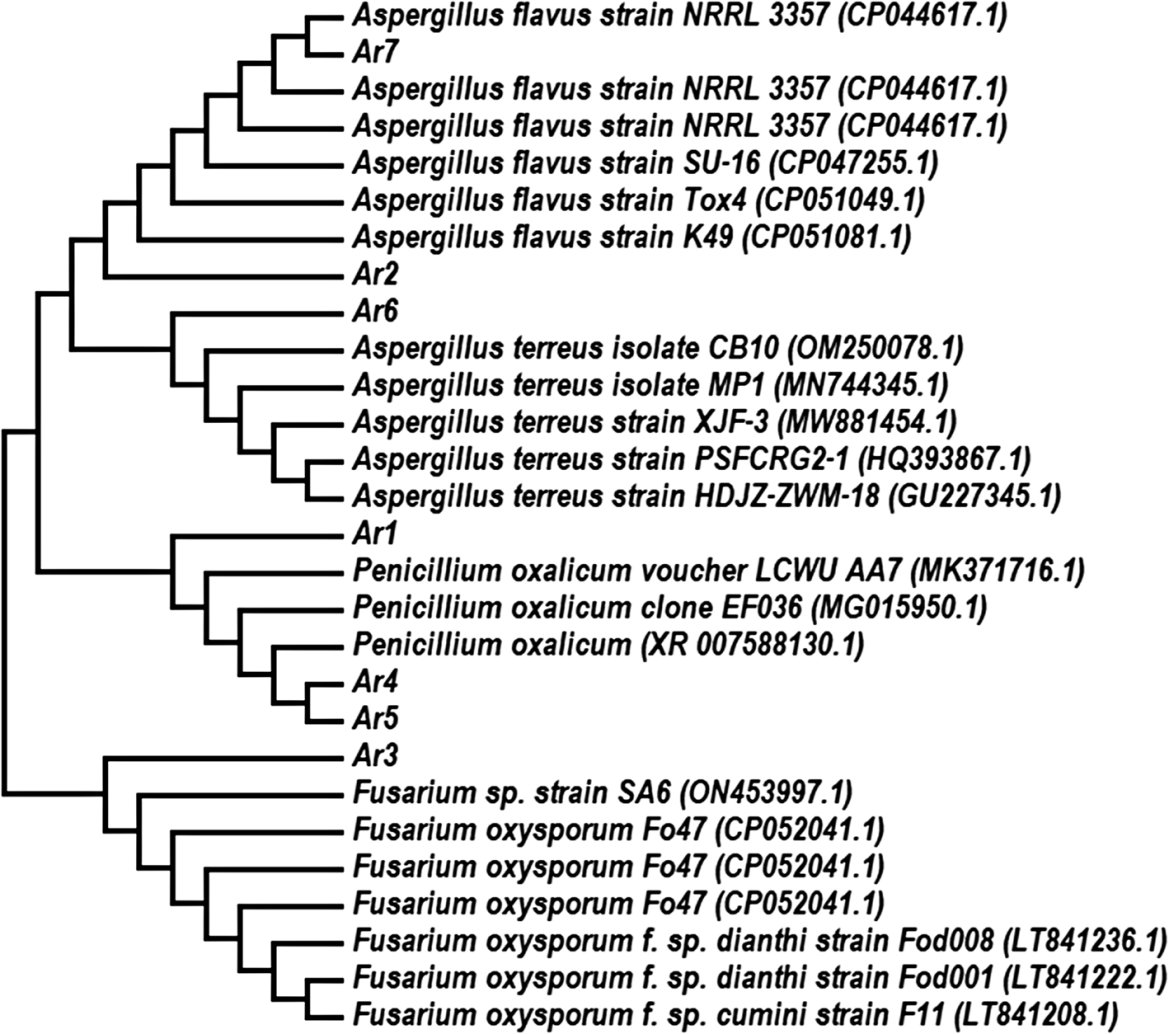
Neighbour-joining tree based on 18 S rRNA gene sequences (-3007.6162 bp length) of the isolated species. The tree included accession numbers in parenthesis and bootstrap values

A phylogenetic tree based on the comparison of 18 S rRNA gene sequences with reference strains was constructed. The phylogenetic analysis was performed with 15,000 and 2000 bp sequences for *Penicillium oxalicum, Fusarium oxysporum*, *Aspergillus flavus*, and *Aspergillus terreus* using the software MEGA 6 as shown in Fig. 2.

**Table 1:** Identification of culturable jute endophytic fungi from seeds

Isolates codes	Close Relatives	Similarity	Coverage	Gene bank Accession no. of closely related strain	
**Ar1**	*Penicillium oxalicum*	99.91%	99%	MG585101.1	
**Ar2**	*Aspergillus flavus*	99.66%	100%	CP051025.1	
**Ar3**	*Fusarium oxysporum*	99.91%	100%	LT841236.1	
**Ar4**	*Penicillium oxalicum*	100.00%	100%	EF411061.1	
**Ar5**	*Penicillium oxalicum*	99.82%	99%	MK371716.1	
**Ar6**	*Aspergillus terreus*	100%	99%	MN995500.1	
**Ar7**	*Aspergillus flavus*	100%	100%	CP051025.1	

### Anti-infective activity screening

The major goal of our study is to establish new sources of antibiotics and other bioactive compounds. It has been reported that endophytes are a source of new leads with new bioactivities; this targeted approach is a productive way to make new discoveries [37]. As shown in Table 2, seven strains were selected for the bioactivity against a panel including clinically relevant of bacterial pathogens including, Gram-positive strains (*Staphylococcus aureus* ATCC 9144, *Methicillin-resistant Staphylococcus aureus* ATCC 33,591 and *Klebsiella quasipneumoniae* ATCC 700,603) and Gram-negative strains (*Escherichia coli* ATCC25922 and *Pseudomonas aeruginosa* ATCC 27,853), The human fungal pathogen *Candida albicans* ATCC 10,231 was measured in 96 plates at a concentration of 500 µg /mL in comparison to a reference compound. The results revealed that *Aspergillus* sp. (Ar6) is the sole isolate that showed antibacterial activity against *E. coli* with IC_50s_ of 1.19 µg/mL in comparison to ciprofloxacin as a positive control (1.5 µg/mL). It also exhibited the highest antifungal activity against *C. albicans* with an IC_50_ of 15.0 µg/mL, in comparison to fluconazole as a reference standard (16 µg/mL). So antibacterial and antifungal in silico studies were performed on it to discuss and prove its potency.

**Table 2:** In vitro antimicrobial activity of the isolated strains crude extracts

IC_50_ (µg /mL)						
Antibacterial						Antifungal
**Sample tested**	***S. aureus***	***P. aeruginosa***	***K.quasipneumoniae***	***MRSA***	***E. coli***	***C. albicans***
Ar 1	> 50	> 50	> 50	> 50	10	> 50
Ar 2	> 50	30	> 50	39.0	5.2	> 50
Ar 3	> 50	> 50	25.0	> 50	5.1	> 50
Ar 4	> 50	28	> 50	> 50	6	> 50
Ar 5	> 50	> 50	> 50	> 50	3	> 50
Ar 6	> 50	> 50	> 50	> 50	1.19	15
Ar 7	> 50	> 50	> 50	> 50	2.5	> 50
Ciprofloxacin	21.56	2.64	123.48	19.15	1.5	nd
Fluconazole	nd	nd	nd	Nd	nd	16

**Ciprofloxacin**, positive antibacterial control 100 μg/mL, **Fluconazole**, positive antifungal control 100 μg/mL

### Metabolomic profiles of the culture extracts

Metabolomics is a technique concerned with providing a chemical fingerprint or an entire chemical profile for a specific organism at specific conditions [25]. Likewise, it plays a vital role in the exploring of novel bioactive metabolites and natural drug discovery; it also helps in improving fungal fermentation methods and manages the isolation of various bioactive compounds [25]. Metabolic profiling based on positive and negative ion mode LC-HR-ESI-MS of the ethyl acetate crude extract of the fungal endophytic *Aspergillus* sp. Ar6 isolated from the Corchorus seeds showed their richness in several classes of metabolites. The dereplication was implemented using DNP database and the resulted features were reduced by applying a chemotaxonomic, resulted in 16 metabolites being dereplicated as alkaloids, peptides and terpenoids, where peptides derivatives were found to predominate.

**Fig. 3:**
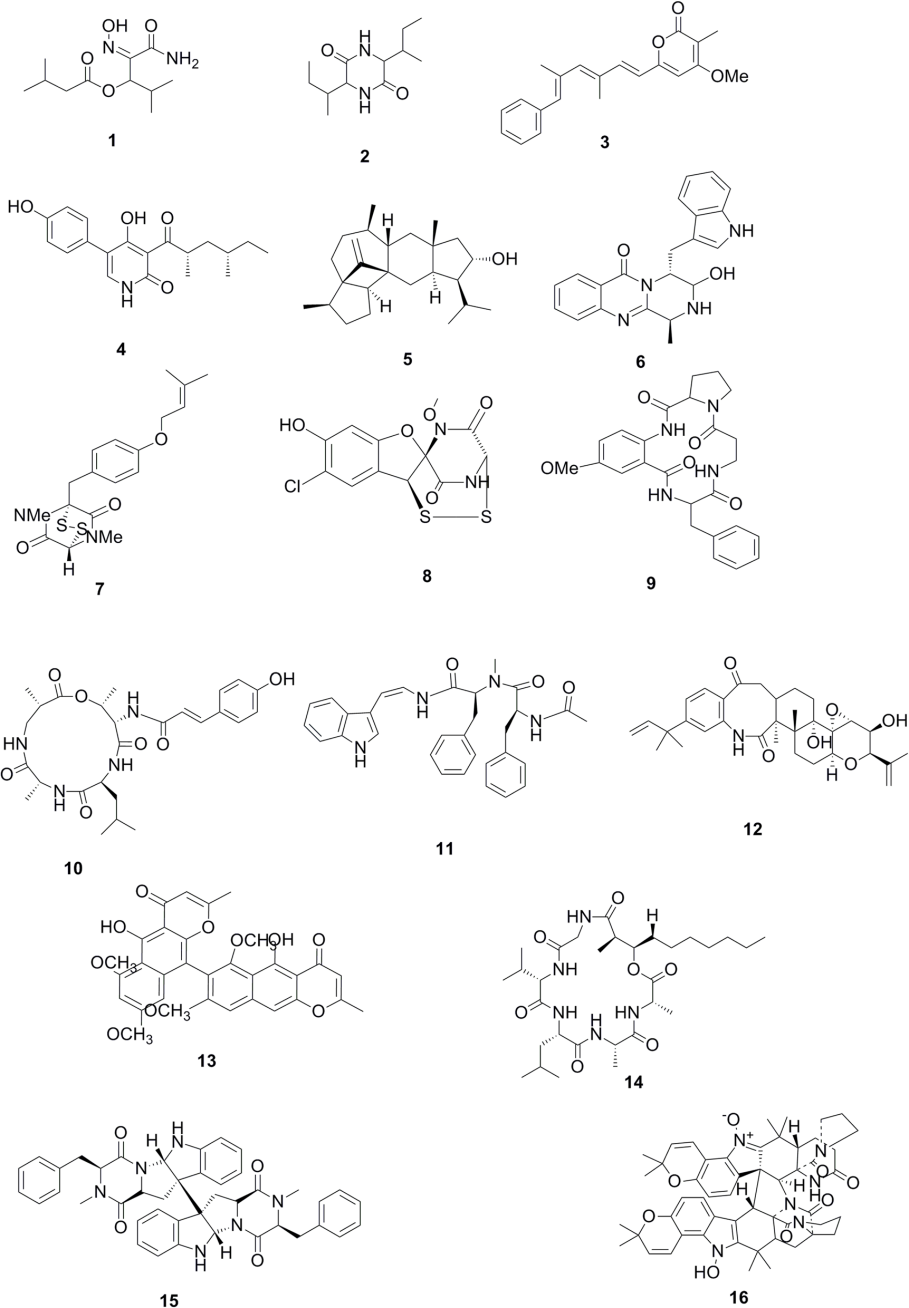
Chemical structures of the *d*ereplicated metabolites of the ethyl acetate crude extract of *Aspergillus* sp. Ar6

The molecular ion mass peak at m/z 245.14 [M^+^H]^+^, for the predicted molecular formulas C_11_H_20_N_2_O_4_ gave hits of the amide type Aspergilliamide B (1), that were previously isolated from marine-derived *Aspergillus westerdijkiae* DFFSC 013. The mass ion peak at m/z 257.14, in consonance with the suggested molecular formula C_12_H_22_N_2_O_4_ [M-H]^−^ was dereplicated as a peptide derivative Cyclo (isoleucylisoleucyl) (2) that were previously isolated from *Aspergillus terreus*. The mass ion peak at m/z 321.14, in conformity with the predicted molecular formula C_21_H_22_O_3_ [M-H]^−^ was dereplicated as α-pyrone derivative, Asnipyrone A (3) previously isolated from *Aspergillus niger* DSM 2182 and MA-132 strains. While the mass ion peak m/z 344.14, corresponding to the suggested molecular formula C_19_H_23_NO_5_ [M-H]^−^ fit an alkaloid compound aspyridone A (4) that were previously isolated from *Aspergillus nidulans*. Another mass ion peak at m/z 355.29 [M-H]^−^, corresponding to the molecular formulas C_25_H_40_O gave hits of the sesterterpenoid compound astellatol (5) that were previously isolated from *Aspergillus variecolor*. Likewise, the mass ion peak at m/z 359.14, corresponding to the suggested molecular formula C_21_H_18_N_4_O_2_ [M + H]^+^ fit an alkaloid compound Fumiquinazoline F (6) that were previously isolated from marine-derived *Aspergillus fumigatus.* The mass ion peak at m/z 377.10, corresponding to the suggested molecular formula C_18_H_22_N_2_O_3_S_2_ [M-H]^−^ fit an epidithiodioxopiperazines compound Dithiosilvatin (7) that was previously isolated from *Aspergillus silvaticus.* The mass ion peak at m/z 422.89, in conformity with the molecular formula C_12_H_9_ClN_2_O_5_S_4_ [M-H]^−^ was identified as aspirochlorine, which is a tetrasulfide analogue compound (8) and previously isolated from *Aspergillus flavus.* The mass ion peak at m/z 463.20, corresponding to the suggested molecular formula C_25_H_28_N_4_O_5_ [M-H]^−^, in accordance with the peptide compound; Aspercolorin (9) that were previously isolated from *Aspergillus versicolor*. Another mass ion peak at m/z 515.25, corresponding to the molecular formula C_26_H_36_N_4_O_7_ [M-H]^−^ in harmony with a peptide analogue compound aspergillipeptide A (10) that was previously isolated from marine-derived *Aspergillus sp.* SCSGAF 0076. The mass ion peak at m/z 525.25 [M + H]^+^ and mass ion peak at m/z 536.29 [M + H]^+^ corresponding to molecular formula C_31_H_32_N_4_O_4_ and C_32_H_41_NO_6_ were identified as Miyakamide A1(11) and Sulpinine C (12) that were previously isolated from *Aspergillus flavus var. columnaris FKI-0739* and *Aspergillus sulphureus*, respectively. As well, the metabolite, namely aurasperone A (13), with the molecular formula C_31_H_28_O_12_, was dereplicated from the mass ion peak m/z 591.15 [M-H]^−^ that was previously isolated from *Aspergillus niger* and *Aspergillus awamori*. In addition, the mass ion peak m/z 596.40 [M + H]^+^, in agreement with the molecular formula C_30_H_53_N_5_O_7_, was dereplicated as Emericellamide C (14) that was previously isolated from *Aspergillus nidulans*. The mass ion peak at m/z 793.34 [M-H]^−^ for the predicted molecular formula C_50_H_46_N_6_O_4_, was dereplicated as Ditryptophenaline (15), that was previously isolated from *Aspergillus flavus*. The mass ion peak at m/z 907.40 [M + H]^+^ for the molecular formula C_52_H_54_N_6_O_9_, was identified as Stephacidin B (16) that was previously isolated from *Aspergillus ochraceus* as shown in Table 3. The chemical structures of the dereplicated metabolites were shown in Fig. 3.

**Table 3:** Metabolites dereplicated from *Aspergillus* sp. Ar6 culture extract

No.	m/z	Rt	MW	Molecular formula	Identifcation	Source	Class	Ref.
1	245.14	7.44	244.142	C_11_H_20_N_2_O_4_	Aspergilliamide B	A. *westerdijkiae*	Amide	[38]
2	257.14	17.69	258.157	C_12_H_22_N_2_O_4_	Cyclo (isoleucylisoleucyl	A. *terreus*	Peptide	[39]
3	321.14	17.04	322.157	C_21_H_22_O_3_	Asnipyrone A	*A. niger*	α-pyrone derivatives	[40]
4	344.14	15.68	345.157	C_19_H_23_NO_5_	Aspyridone A	A. *nidulans*	Alkaloids	[41]
5	355.29	20.05	356.307	C_25_H_40_O	Astellatol	A. *variecolor*	Ses-terterpenoid	[42]
6	359.14	23.98	358.142	C_21_H_18_N_4_O_2_	Fumiquinazoline F	*A. fumigatus*	Alkaloids	[43]
7	377.10	14.09	378.107	C_18_H_22_N_2_O_3_S_2_	Dithiosilvatin	*A. silvaticus*	Epidithiodioxopiperazines	[44]
8	422.89	27.27	423.907	C_12_H_9_ClN_2_O_5_S_4_	Aspirochlorine	*A. flavus*	Epidithiodiketopiperazines	[45]
9	463.20	19.44	464.207	C_25_H_28_N_4_O_5_	Aspercolorin	*A. versicolor*	Peptide	[46]
10	515.25	15.67	516.257	C_26_H_36_N_4_O_7_	Aspergillipeptide A	*Aspergillus* sp.	Peptides	[47]
11	525.25	24.05	524.242	C_31_H_32_N_4_O_4_	Miyakamide A1	*A. flavus* *var. columnaris*	Peptides	[48]
12	536.29	22.65	535.292	C_32_H_41_NO_6_	Sulpinine C	*A. sulphureus*	Alkaloids	[49]
13	591.15	21.64	592.157	C_31_H_28_O_12_	Aurasperone A	*A. niger* and *A. awamori*	Naphtho- pyranones	[50]
14	596.40	23.67	595.392	C_30_H_53_N_5_O_7_	Emericellamide C	*A. nidulans*	Cyclo-peptides	[51]
15	793.34	25.29	794.357	C_50_H_46_N_6_O_4_	Ditryptophenaline	*A. flavus*	Peptide	[52]
16	907.40	21.78	906.392	C_52_H_54_N_6_O_9_	Stephacidin B	*A. ochraceus*	Alkaloids	[53]

### *In silico* biological activity predictions

*In-silico* studies have effectively integrated with experimental research to explain complicated chemical and biological processes. Combining computational and experimental techniques has proven highly beneficial in identifying the potential targets of biologically active compounds and their mechanism of action [54]. We used computational tools to investigate the principal components of the *Aspergillus* sp. Ar6 crude extract that were responsible for exhibition its antimicrobial activity against *E. coli* and *C. albicans*, and to predict their mechanism of action. According to a literature survey, certain bioactive compounds of the extract were reported to have antibacterial or antifungal activities against *E. coli* and *C. albicans*, respectively as shown in (Supplementary materials, Table S1). The remaining unknown-activity compounds were applied to statistical screening of antimicrobial activity. These compounds were submitted to the PASS Online web server for the prediction of antibacterial and antifungal activities against different microbial species, and the results listed in (Supplementary materials, Table S2). The results revealed the high probability for Aspergillipeptide A (10), and Emericellamide C (14) to have antibacterial and antifungal activities (Pa > 0.5). Also, Asnipyrone A (3), and Astellatol (5) showed Pa score higher than 0.5 for antifungal activity only. While other compounds have no possibility to act as antibacterial or antifungal agents (Pa < 0.5).

After filtering the results, the selected compounds Aspergillipeptide A (10) and Emericellamide C (14) were applied for AntiBac-pred and AntiFun-pred tools to investigate which could exert antimicrobial activity against *E. coli* and *C. albicans*, respectively [33]. On the other side, compounds Asnipyrone A (3) and Astellatol (5) were applied for AntiFun-pred only to determine its activity act against *C. albicans* (Supplementary materials, Table S3). Aspergillipeptide A (10) and Emericellamide C (14) were predicted to be active against *E. coli* and *C. albicans*. In addition, Astellatol (5) was predicted to be active against *C. albicans* while Asnipyrone A (3) was predicted to have no effect on *C. albicans.*

### Prediction of the potential targets

#### Prediction of the potential *E. coli* targets of the annotated compounds

##### Construction of PPI network

Considering the importance of identifying the therapeutic targets in which different phytochemicals act as antimicrobial and antifungal, PharmMapper server used for identifying potential targets for the given compounds using pharmacophore mapping approach [55]. PharmMapper was employed to predict targets for Aspergillipeptide A (10) and Emericellamide C (14). We received 300 protein targets for each compound from the PharmMapper result list (Supplementary materials, Table S4-S5). After analyzing results, only 28 bacterial-related proteins found in *E. coli* organism were collected for Aspergillipeptide A (10), while 27 bacterial targets were found for Emericellamide C (13) (Supplementary materials, Table S6).

**Fig. 4:**
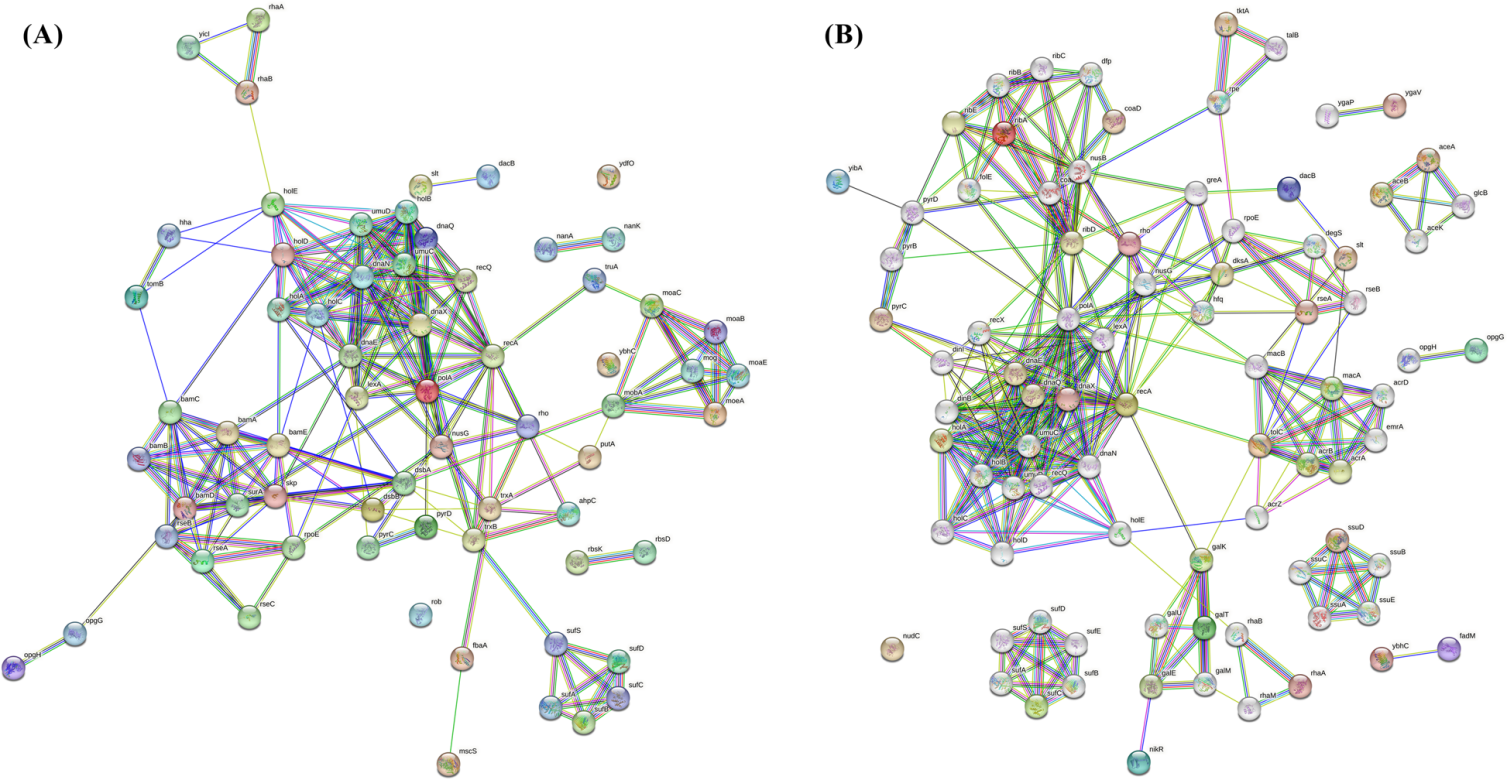
PPI network constructed by STRING. **A** PPI network for Aspergillipeptide A related targets; The PPI network consists of 66 nodes and 219 edges. **B** PPI network for Emericellamide C related targets; The PPI network consists of 85 nodes and 296 edges

After filtration results, the related proteins for each compound were uploaded to the STRING database 11.5 to construct the primary PPI networks and find their direct and functional partners. Figure 4 illustrates the PPI networks for targets related to Aspergillipeptide A (10) and Emericellamide C (14). Each edge in the network represented a protein interaction and each node represented a target.

The related proteins for each compound underwent enrichment analysis to understand their biological functions and possible pathways. Enrichment analyses were performed through Gene Ontology (GO) and Kyoto Encyclopedia of Genes and Genomes (KEGG).

In addition, PPI network was then exported to the software Cytoscape 3.9.1 which was utilized to analyze the network using CytoHubba plugin. CytoHubba plugin calculated, through 12 kinds of methods, the most significant and coinciding nodes which would be potential targets for the related compounds. The interactions and binding modes between selected compounds and filtered targets were examined through molecular docking.

##### Gene ontology (GO) enrichment analysis

**Fig. 5:**
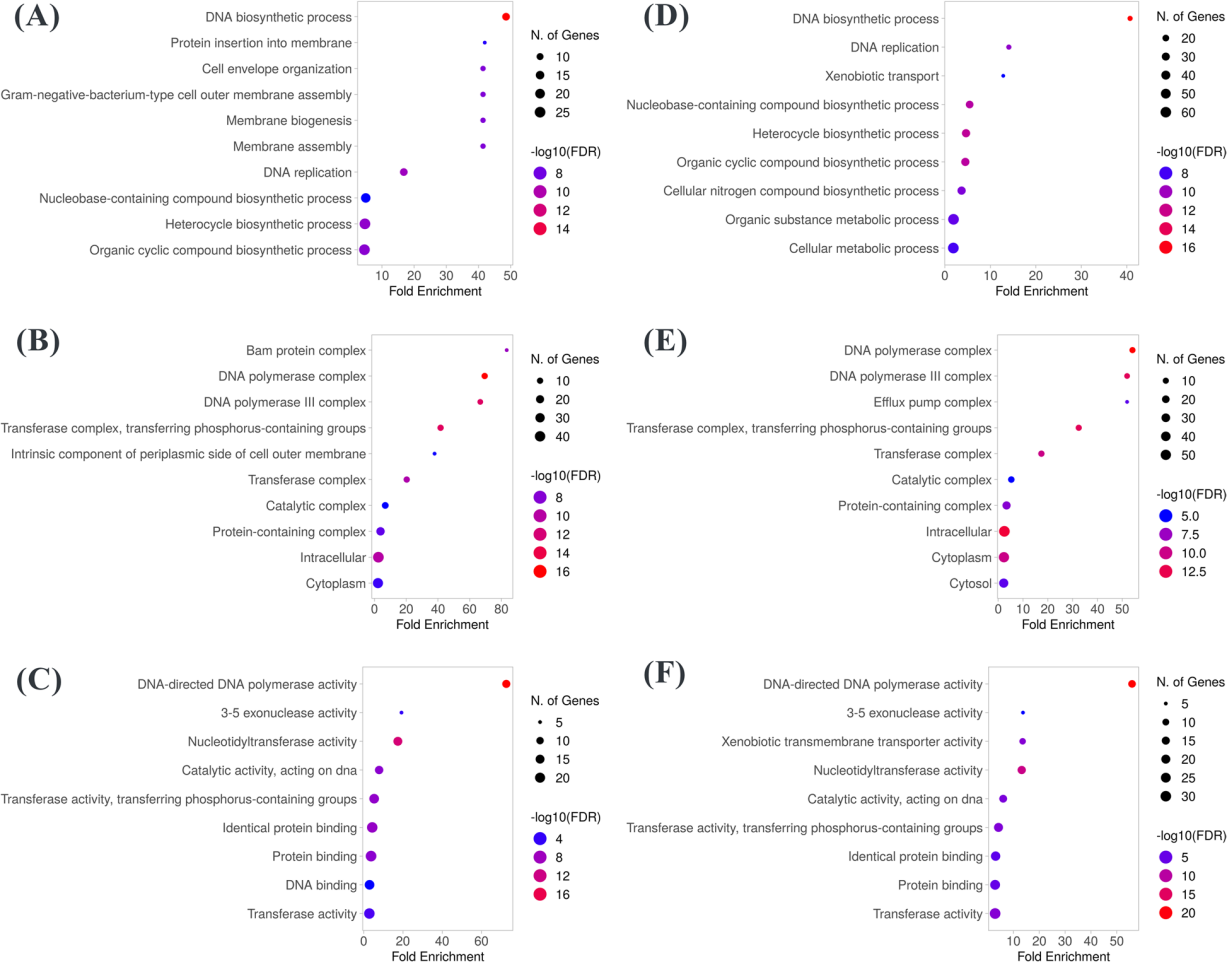
GO enrichment analysis. **A**-**C** BB, CC, and MF of target proteins related to Aspergillipeptide A (10). **D**-**F** BB, CC, and MF of target proteins related to Emericellamide C (14). The intensity of the color represents the log function of corrected p-value (FDR) (FDR < 0.05). The higher the enrichment score and the redder the color, the more significant the function was

GO enrichment analysis is a method for interpreting sets of genes or genes products into their biological functions including biological processes (BB), cellular components (CC), and molecular functions (MF) [56]. The collected proteins were converted to the corresponding uniport identifiers and were inserted into ShinyGO 0.77 [57]. The top 10 significantly enriched results in each category of GO analysis are shown in Fig. 5.

The BB primarily involved two main processes (Fig. 5, A). The first process was DNA replication which is represented by the most enriched process, DNA biosynthetic process (GO:0071897). Also, other processes such as DNA replication (GO:0006260), nucleobase-containing biosynthetic process (GO:0034654), heterocycle biosynthetic process (GO:0018130), and organic cyclic biosynthetic process (GO:1,901,362) are the highest in genes number and are also related to DNA biosynthesis. The other biological process is the membrane biogenesis and organization which is represented by membrane biogenesis (GO:0044091), membrane assembly (GO:0071709), gram-negative-bacterium-type cell outer membrane assembly (GO:0043165), and cell envelope organization (GO:0043163). While the most significant GO items related to CC mainly included DNA polymerase complex (GO:0042575), DNA polymerase III complex (GO:0009360) (Fig. 5, B). These cellular components play a vital role in DNA replication. In addition, DNA-directed DNA polymerase activity (GO:0003887) is the most significantly enriched term in MF (Fig. 5, C). While other important MF items were nucleotidyltransferase activity (GO:0016779), and catalytic activity, acting on DNA (GO:0140097).

In case of GO analysis for Emericellamide C (14), BP were also dominated by DNA biosynthetic process (GO:0071897), DNA replication (GO:0006260), and their other related process such as nucleobase-containing biosynthetic process (GO:0034654), heterocycle biosynthetic process (GO:0018130), and organic cyclic biosynthetic process (GO:1,901,362) (Fig. 5, D). Moreover, DNA polymerase complex (GO:0042575) and DNA polymerase III complex (GO:0009360) are the most prominent items in CC (Fig. 5, E). MF enrichment results included DNA-directed DNA polymerase activity (GO:0003887) as same as results of Aspergillipeptide A (Fig. 5, F). All these GO results indicated that the action of Aspergillipeptide A (10) and Emericellamide C (14) would be through interfering DNA metabolic process, especially by inhibiting DNA polymerase activity.

##### KEGG pathway enrichment analysis

**Fig. 6:**
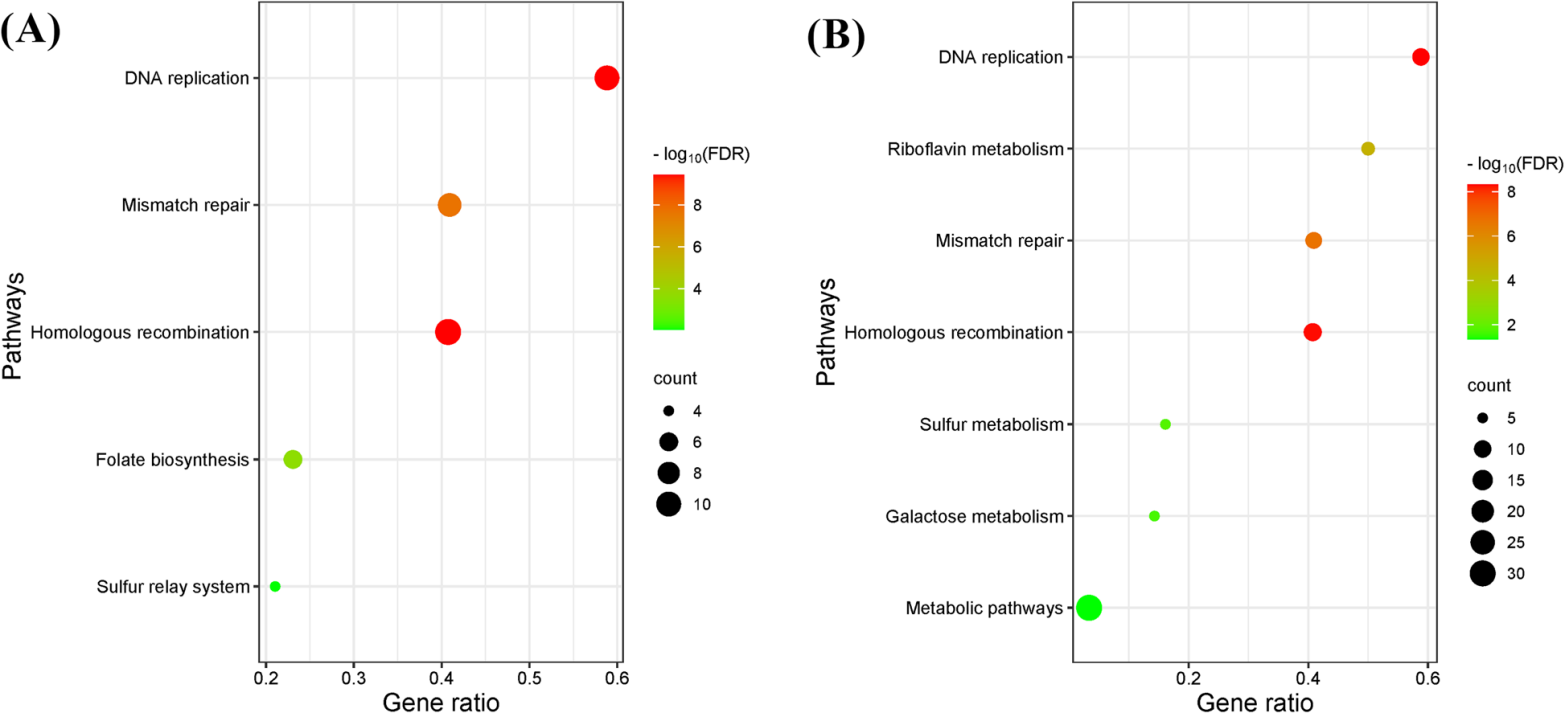
KEGG pathway enrichment analysis. **A** KEGG pathways enrichment of target proteins of Aspergillipeptide A (10). **B** KEGG pathways enrichment of target proteins of Emericellamide **C** (14). The x-axis represents gene ratio (number of genes enriched in the pathway/number of all genes in the background gene set) and the y-axis represents pathways. The color of the dot represents enrichment significance, and its size indicates the number of genes enriched in the pathway

The KEGG enrichment analysis is the process to mapping protein targets to their molecular pathways [58]. KEGG enrichment results showed the possible pathways involved in the action of the protein targets related to selected compounds and the top 10 results were selected using enrichment score Fig. 6. KEGG analysis showed that Aspergillipeptide A (10) and Emericellamide C (14) related proteins would be involved in DNA replication, homologous recombination, mismatch repair, as the most significant pathways. Also, riboflavin metabolism is one of the important pathways represented for target proteins of Emericellamide C (14).

This high similarity of GO and KEGG enrichment analyses between Aspergillipeptide A (10) and Emericellamide C (14) derived us to explore their structural similarity and flexible alignment.

##### Flexible alignment study

**Fig. 7:**
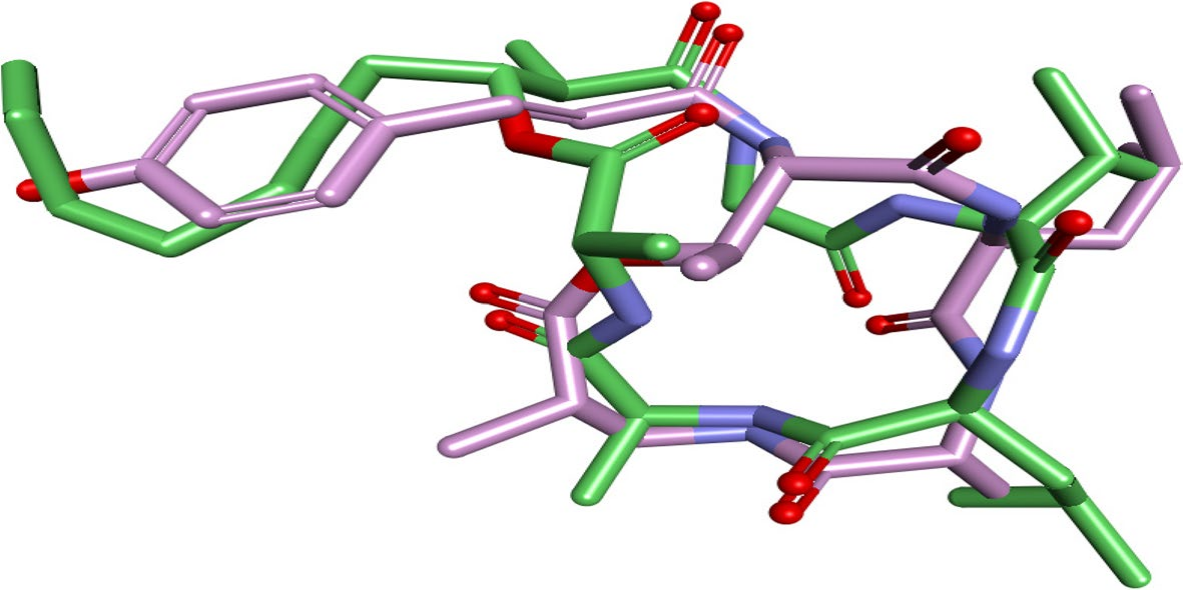
Flexible alignment of Aspergillipeptide A (**10**) and Emericellamide C (14). Aspergillipeptide A (10) is colored violet and Emericellamide C (14) is colored green

Flexible alignment is a computational procedure used for superposing small organic molecules for the assessment of their 3D similarity. The flexible alignment was constructed using Aspergillipeptide A and Emericellamide C to investigate the 3D structural similarity against each other through generation of the lowest energy each compound, and application of molecular overlay tool in Discovery Studio. Figure 7 showed that Aspergillipeptide A and Emericellamide C were good aligned with overlay similarity of 0.6066. Two molecules have a similar size and shape and their hydrophilic areas, and hydrophobic areas are overlapped. This explains the resemblance of the GO & KEGG analysis between Aspergillipeptide A and Emericellamide C.

##### Identification of potential protein targets

The CytoHubba plugin was utilized to calculate the 12 different metrics (EPC, MCC, DMNC, MNC, Closeness, Degree, BottleNeck, Betweenness, Stress, EcCentricity, Radiality, and Clustering Coefficient) of all nodes for each PPI network. After calculating the 12 centrality measures of all nodes (Supplementary materials, Table S7), the top 10 nodes of each measure were collected. Moreover, the number of occurrences for overlapping nodes were calculated and these nodes were then sorted in a descending order as shown in the Figure S1. In case of Aspergillipeptide A (10) related-PPI network, the nodes (dnaE, PolA, recA, dnaX) had the highest number of occurrence (Figure S1, A), which indicates DNA polymerase III subunit alpha, DNA polymerase I, DNA recombination protein, and DNA polymerase III subunit tau, respectively. While (recA, PolA, ribD, dnaX, umuC, and holB) nodes are the most coinciding nodes in Emericellamide C (14) related-PPI network (Figure S1, B). These nodes indicate DNA recombination protein, DNA polymerase I, riboflavin biosynthesis protein, DNA polymerase III subunit tau, mutagenesis protein UmuC, and DNA polymerase III subunit delta’. Nodes which were considered as potential targets were considered for the further molecular docking study.

#### Prediction of the potential *C. albicans* targets of the annotated compound

For the investigation of antifungal targets against *C. albicans*, only 3 targets were recognized as the potential targets after analyzing results from PharmMapper. The annotated compounds (5, 10, and 14) were probably showed pharmacophore matching with geranylgeranyltransferase-I (PDB ID: 3DRA), secreted aspartic proteinase (Sap) 5 (PDB ID: 2QZX), and *N*-acetylglucosamine-phosphate mutase (PDB ID: 2DKC). These targets are contributing in essential processes for fungal viability, virulence, and pathogenesis [59–61]. The three targets ranked by normalized fit score in descending order for each compound Table 4. Also, the interactions between compounds and these fungal targets were investigated through molecular docking.

**Table 4:** * C. albicans* protein targets for the selected compounds extracted from PharmMapper server and ranked by normalized fit score in descending order

PDB ID	Normalized fit score		
	Astellatol (**5**)	Aspergillipeptide A (**10**)	Emericellamide C (**14**)
3DRA	0.3525	0.2493	0.2418
2QZX		0.2684	0.3628
2DKC		0.1976	0.1967

‘-’ means not found

### Molecular docking results

#### Molecular docking results for the antibacterial study

The core targets were selected for molecular docking using AutoDock 4.2.6 to verify their binding affinity with Aspergillipeptide A (10) and Emericellamide C (14). Aspergillipeptide A (10) and Emericellamide C (14) were docked with *E. coli* DNA recombination protein (RecA) (PDB ID: 3CMT) and *E. coli* DNA polymerase III (Pol III) (PDB ID: 2NHN). Emericellamide C (14) was also investigated for interactions with bifunctional deaminase/reductase (RibD) of the riboflavin biosynthetic pathway (PDB ID: 2OBC). The docking analysis results are presented in Table 5.

**Table 5:** Docking scores of the annotated compounds with three potential bacterial targets

	Binding energy (Kcal/mol)		
Compounds	RecA	Pol III	RibD
Aspergillipeptide A (**10**)	-7.15	-5.72	
Emericellamide C (**14**)	-6.98	-8.23	-7.85

‘-’ means not measured

**Fig. 8:**
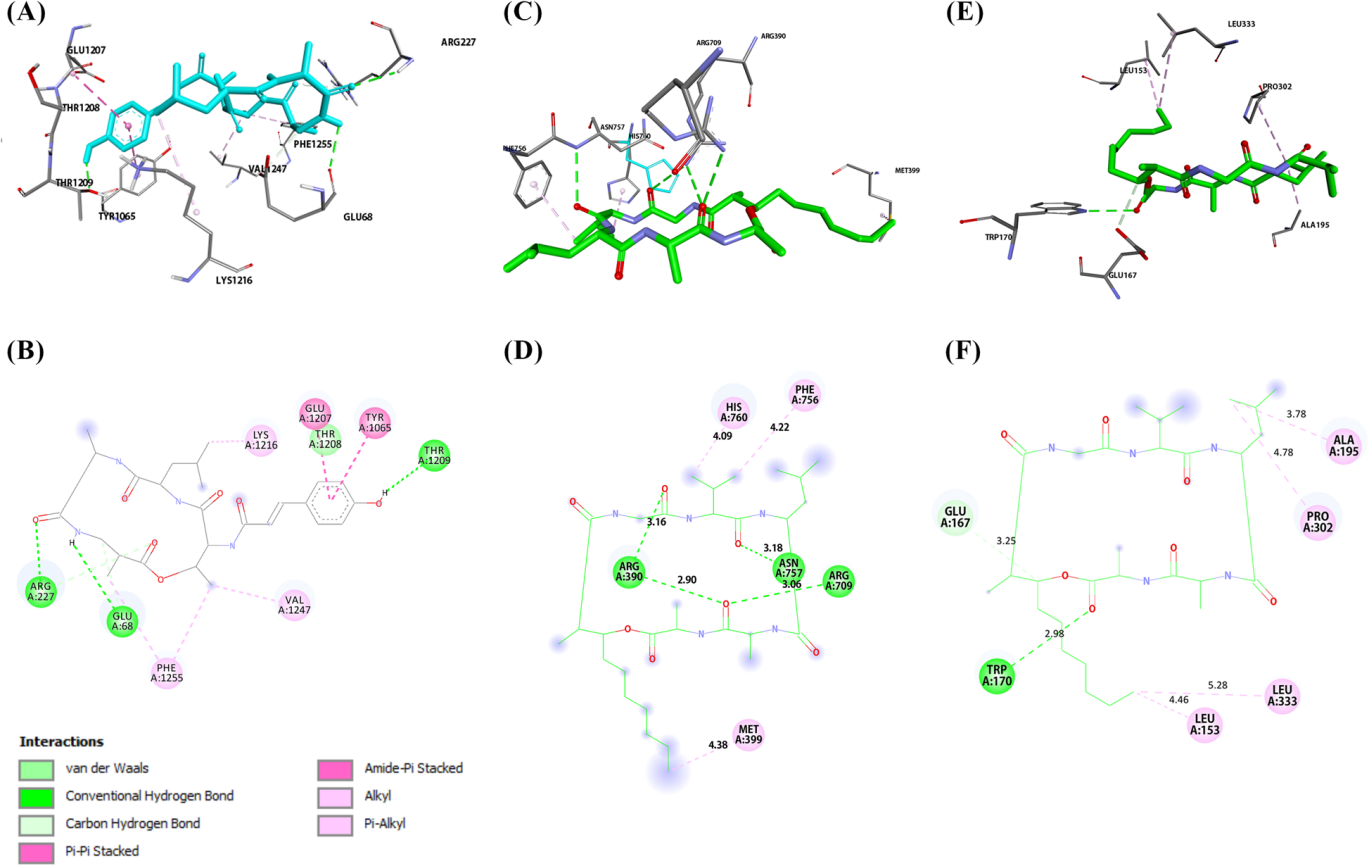
**A** 3D binding mode of Aspergillipeptide A with E. coli DNA recA (PDB ID: 3CMT), (**B**) 2D diagram for binding interactions of Aspergillipeptide A with E. coli DNA recA. **C** 3D binding mode of Emericellamide C with *E. coli* DNA Pol III (PDB ID: 2HNH). **D** 2D diagram for binding interactions of Emericellamide C with *E. coli* DNA Pol III. **E** 3D binding mode of Emericellamide C with *E. coli* ribD (PDB ID: 2OBC). **F** 2D diagram for binding interactions of Emericellamide C with *E. coli* ribD

Aspergillipeptide A (10) exhibited the highest binding affinity for *E. coli* DNA RecA with binding energy equals − 7.15 Kcal/mol. As shown in (Fig. 8A and B), Aspergillipeptide A forms H-bonds and hydrophobic interactions with the amino acids of the active site. These values indicate that Emericellamide C (14) has a strong binding affinity for Pol III and RibD, but a slightly weaker binding affinity for RecA compared to Aspergillipeptide A (10). While Emericellamide C (14) was able to interact with DNA polymerase III active site through three hydrogen bonds (Fig. 8C and D).

The *E. coli* RecA monomer has three major structural domains: a small N-terminal domain (NTD), a central domain and a large C-terminal domain (CTD). The central domain contains the ATP binding site, which is essential for the catalytic activity of the protein [62]. Aspergillpeptide A (10) has shown to interact with ATP binding site through H-bonds with GLN68, ARG227, and THR1209. Moreover, phenyl group of Aspergillpeptide A showed interactions with TYR1065, GLU1207.

In *E. coli*, the replicative polymerase is DNA Pol III, which is composed of 10 different subunits that work together to coordinate the replication of both the leading and lagging strands of DNA. Recent research has identified six essential amino acid residues, specifically ARG362, ASP405, LYS553, TYR686, GLN688, and HIS760, for the activity of Pol III. In addition, three other important amino acid residues, TYR340, ARG390, and LYS758, also play crucial roles in the activity of Pol III [63]. Molecular docking analysis revealed the ability of carbonyl groups of Emericellamide C (14) to bind with key amino acid ARG390 through two H-bonds. Also, aliphatic side chains made hydrophobic interactions with MET399, HIS760, PHE756. It was observed repulsive interactions between Aspergillipeptide A (10) and (ARG709, GLN687) amino acids, which could be reasonable for the low binding energy of Aspergillipeptide A (**10**) with DNA Pol III.

RibD is one of crucial proteins that participate in riboflavin biosynthesis which is essential to bacterial growth. Hence, inhibition of ribD is considered as antibacterial drug target. RibD contains two separate domains for deamination and reduction. The reductase active site is lined by the loop GLY159–SER173, which makes important interactions with the cofactor (NADP^+^) and substrate analogue in the binary complexes. The NADP^+^ nicotinamide ring makes a 3.5 Å pi-pi stacking interaction with Trp170. [64] Emericellamide C (14) showed stronger interaction with Trp170 than the cofactor with binding affinity − 7.85 Kcal/mol (Fig. 8, E and F). Also, Emericellamide C (14) formed hydrophobic interactions with LEU135, ALA195, PRO302, and LEU333.

#### Molecular docking results for the antifungal study

**Fig. 9:**
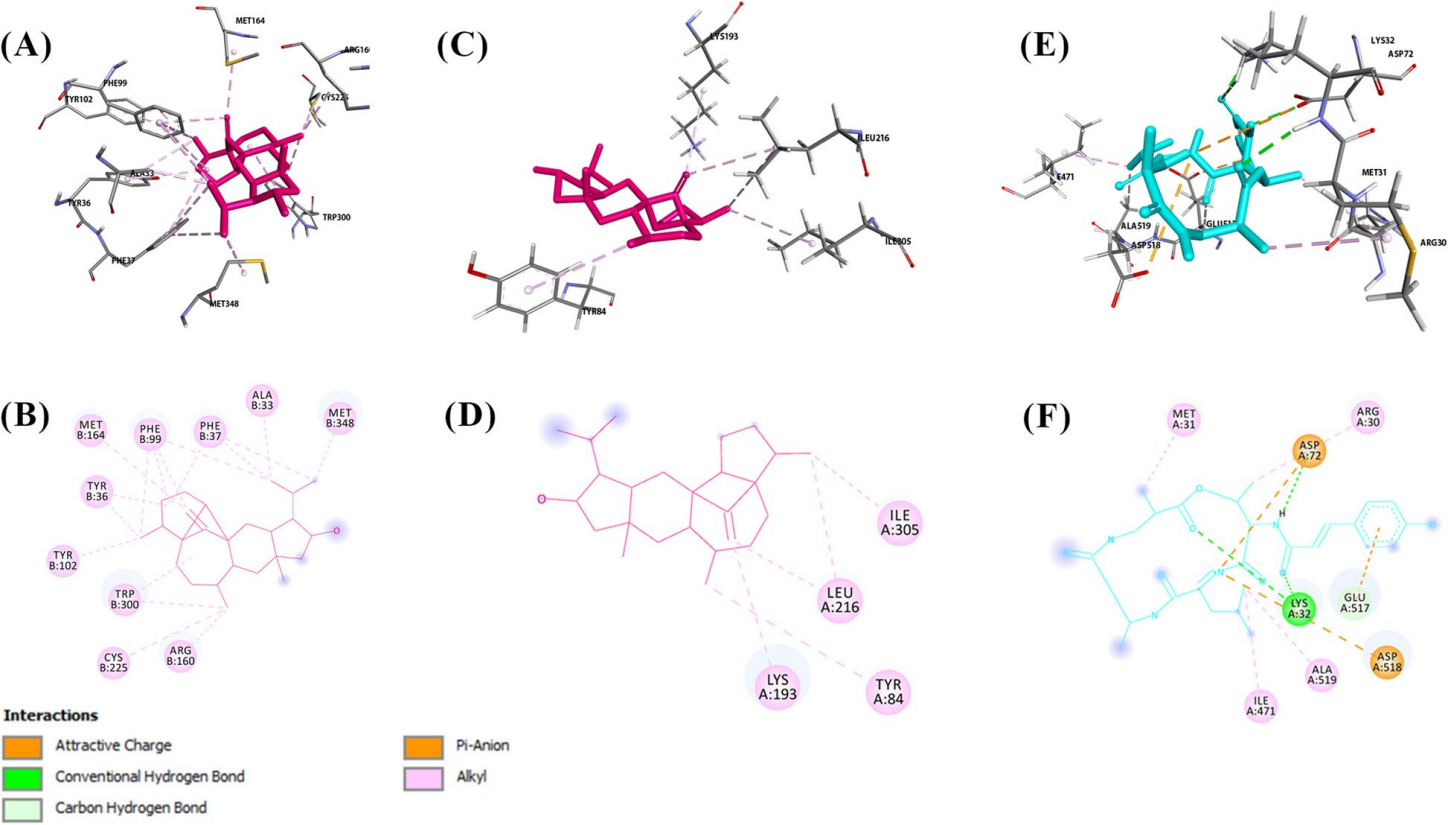
**A** 3D binding mode of Astellatol with geranylgeranyltransferase-I (PDB ID: 3DRA), (**B**) 2D diagram for binding interactions of Astellatol with 3DRA. **C** 3D binding mode of Astellatol with Sap5 (PDB ID: 2QZX). **D** 2D diagram for binding interactions of Astellatol with Sap5. **E** 3D binding mode of Aspergillipeptide A with *N*-acetylglucosamine-phosphate mutase (PDB ID: 2DKC). **F** 2D diagram for binding interactions of Aspergillipeptide A with 2DKC

The molecular docking was also carried out for annotated compounds (5, 10, and 14) to be tested against the three fungal targets using AutoDock 4.2.6. The docking analysis results, presented in Table 6, revealed the high affinity of Astellatol (5) towards geranylgeranyltransferase-I and Sap5. In 3DRA active site, Astellatol (5) was found to be stabilized by a high number of hydrophobic interactions with amino acids as shown in Fig. 9A-B. Also, Astellatol (5) showed a stable binding interaction with Sap5 residues (TYR84, LYS193, LEU216, ILE305) through hydrophobic bonds (Fig. 9, C and D). For docking results against *N*-acetylglucosamine-phosphate mutase (2DKC), Emericellamide C (14) has the lowest binding energy with *N*-acetylglucosamine-phosphate mutase, while Aspergillipeptide A (10) was found to be the highest binding amongst other molecules with − 8.15 Kcal/mol. The carbonyl groups of the compound 10 created H-bonds with LYS32 and ASP72 (Fig. 9, E and F). In addition, phenolic group of 10 formed pi-anion bond with GLU517, while aliphatic side chains formed hydrophobic bonds with ARG30, MET31, ILE471, and ALA519.

**Table 6:** The binding energy of the selected compounds for antifungal activity

PDB ID	Binding energy (Kcal/mol)		
	Astellatol (**5**)	Aspergillipeptide A (**10**)	Emericellamide C (**14**)
3DRA	-9.11	-8.11	-8.12
2QZX	-8.03	-7.75	-7.02
2DKC	-7.93	-8.15	-6.92

## Conclusion

**Fig. 10:**
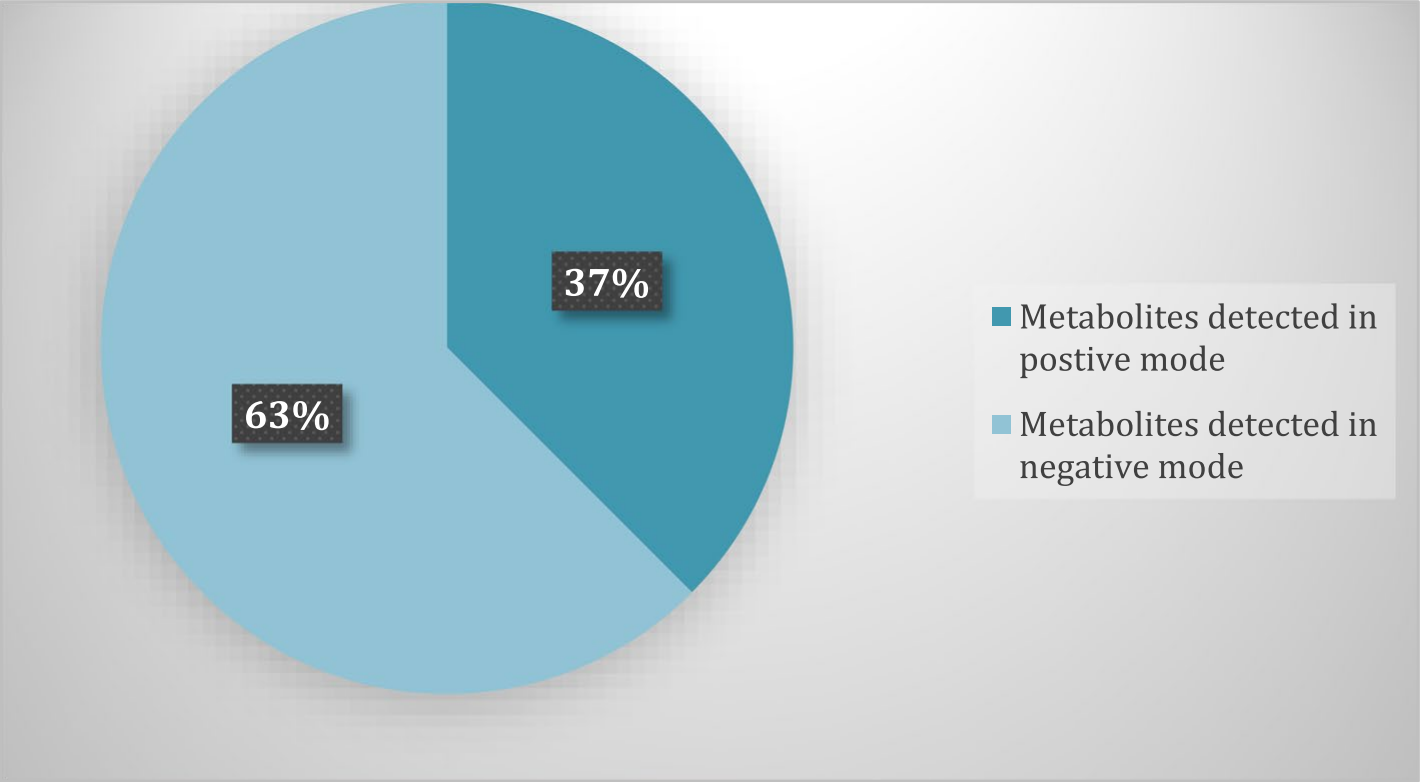
Metabolites detected using LC-HR-ESI-MS profiling in positive and negative mode

**Fig. 11:**
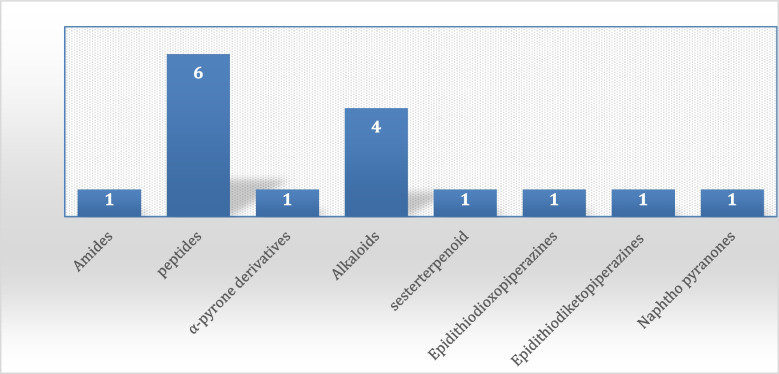
Distribution of metabolites (divided in chemical classes) isolated from Aspergillus sp. (Ar6)

The growing spread of infectious diseases caused by microbes such as bacteria, viruses, and fungi has become one of the most significant challenges for the future of humanity. Plant Endophytes have become an important source of pharmacologically active metabolites. The chemical annotation of *Aspergillus* sp. Ar6 using LC-HR-ESI-MS revealed their richness in diverse metabolites and 16 compounds were annotated in positive and negative modes (Fig. 10**)**, belonging to different classes such as alkaloids, peptides and terpenoids based natural compounds, where peptide derivatives were found to be more predominate Fig. 11. *In silico* screening of the dereplicated metabolites in the fungal extract revealed several compounds that could be potentially active entities. It was further proposed that these active compounds have antibacterial and antifungal activity. A combination of metabolomics and *in-silico* approaches have allowed a shorter route to search for antibacterial and antifungal natural products in a shorter time. The *in-silico* studies for target prediction of antibacterial compounds showed the ability of Aspergillipeptide A to interact with recA and the role of Emericellamide C in inhibiting the action of DNA Pol III and ribD targets. For target prediction of antifungal compounds, the result indicates that Astellatol could bind to two potential targets (geranylgeranyltransferase-I and Sap5), and Asperagillipeptide A could bind to *N*-acetylglucosamine-phosphate mutase. The accurate mechanism in view of molecular docking also demands further validation in biological experiments. In conclusion, the diversity of fungal endophytes isolated from *Corchorus olitorius* seeds has now been reported for the first time. According to these findings, *C. olitorius* is home to a diverse fungal endophytic community with antibacterial properties. A powerful antibacterial extract from *Aspergillus* sp. Ar6 was discovered to be a source of novel antimicrobial chemicals that may be used in the creation of pharmaceuticals.

### Supplementary Information

**Additional file 1: Table S1. **Reported antimicrobial activity of certain compounds of Aspergillus terreus extract against E. coli and C. albicans.  **Table S2.** PASS prediction scores for the unknown-activity compounds as antibacterial and antifungal agents. **Table S3.**AntiBac-pred and AntiFun-pred confidence scores for the selected compounds against E. coli and C. albicans from PASS online analysis. **Table S4.**Results of PharmMapper server forAspergillipeptide A (**10**) ranked by normalized fit score in descending order.** Table S5. **Results of PharmMapper server forEmericellamide C (**14**) ranked by normalized fit score in descending order.** Table S6. **E. coli protein targets for Aspergillipeptide A (10) and Emericellamide C (14) extracted from PharmMapper server and ranked by normalized fit score in descending order. **Table S7. **Results of top 10 nodes calculated by 12 different centrality measures for PPI networks of Aspergillipeptide A (**10**) and Emericellamide C (**14**). **Figure S1. **Number of occurrences for coinciding nodes from PPI networks. (A) Coinciding nodes of PPI network related to Aspergillipeptide A (**10**). (B) Coinciding nodes of PPI network related to Emericellamide C (**14**).

## Data Availability

All data generated or analyzed during this study are included in this article (and its supplementary information files).
