# Supplementary material for: Evaluation of the anti-infective potential of the seed endophytic fungi of Corchorus olitorius through metabolomics and molecular docking approach
Source: BMC Microbiol. 2023 Nov 18;23:355. doi: 10.1186/s12866-023-03092-5 (PMC10656998; doi:10.1186/s12866-023-03092-5)
Supplement: Supplementary file 1 — Additional file 1: Table S1. Reported antimicrobial activity of certain compounds of Aspergillus terreus extract against E. coli and C. albicans. Table S2. PASS prediction scores for the unknown-activity compounds as antibacterial and antifungal agents. Table S3.AntiBac-pred and AntiFun-pred confidence scores for the selected compounds against E. coli and C. albicans from PASS online analysis. Table S4.Results of PharmMapper server forAspergillipeptide A (10) ranked by normalized fit score in descending order. Table S5. Results of PharmMapper server forEmericellamide C (14) ranked by normalized fit score in descending order. Table S6. E. coli protein targets for Aspergillipeptide A (10) and Emericellamide C (14) extracted from PharmMapper server and ranked by normalized fit score in descending order. Table S7. Results of top 10 nodes calculated by 12 different centrality measures for PPI networks of Aspergillipeptide A (10) and Emericellamide C (14). Figure S1. Number of occurrences for coinciding nodes from PPI networks. (A) Coinciding nodes of PPI network related to Aspergillipeptide A (10). (B) Coinciding nodes of PPI network related to Emericellamide C (14). [file 12866_2023_3092_MOESM1_ESM.docx]

**Evaluation of the anti-infective potential of the seed endophytic fungi of *Corchorus olitorius* through metabolomics and Molecular Docking approach**

Arwa Mortada Ahmed^1^, Ayman M. Ibrahim^2^, Ramadan Yahia^3^, Nourhan Hisham Shady,^1^ Basma Khalaf Mahmoud^4^, Usama Ramadan Abdelmohsen^1,4^, Mostafa Ahmed Fouad^4^

^1^ Department of Pharmacognosy, Faculty of Pharmacy, Deraya University, 61111 New Minia City, Egypt

^2^Department of Pharmaceutical Chemistry, Faculty of Pharmacy, Deraya University,61111 New Minia, Egypt

^3^ Department of Microbiology and Immunology, Faculty of Pharmacy, Deraya University, New Minia City, Minia, Egypt

^4^ Department of Pharmacognosy, Faculty of Pharmacy, Minia University, 61519 Minia, Egypt

**Table S1.** Reported antimicrobial activity of certain compounds of Aspergillus terreus extract against E. coli and C. albicans.

| Compound | Published IC_50_ | Ref. |
| --- | --- | --- |
| Asnipyrone A (**3**) | No activity aganist *E. coli* | [31] |
| Fumiquinazoline F (**6**) | > 100^a^ (*E. coli*) | [49] |
| Aspirochlorine (**8**) | No activity aganist *E. coli*  0.028^b^ (*C. albicans*) | [50]  [46] |
| Aurasperone A (**13**) | 8.52^a^ (*E. coli ATCC 35218*)  4.26^a^ (*E. coli* ATCC 25922)  No activity aganist *C. albicans* | [47]  [47]  [51] |

^a^ IC_50_ unit is mg/ml.

^b^ IC_50_ unit is µM.

**Table S2.** PASS prediction scores for the unknown-activity compounds as antibacterial and antifungal agents*.*

| No. | Compound | Antibacterial | | Antifungal | |
| --- | --- | --- | --- | --- | --- |
|  |  | Pa | Pi | Pa | Pi |
| 1 | Aspergilliamide B (**1**) | 0.402 | 0.029 | 0.404 | 0.049 |
| 2 | Cyclo(isoleucylisoleucyl) (**2**) | 0.425 | 0.025 | 0.476 | 0.035 |
| 3 | Asnipyrone A (**3**) | - | - | 0.647 | 0.014 |
| 4 | Aspyridone A (**4**) | 0.374 | 0.037 | 0.475 | 0.035 |
| 5 | Astellatol (**5**) | 0.488 | 0.018 | 0.606 | 0.018 |
| 6 | Fumiquinazoline F (**6**) | - | - | 0 | 0 |
| 7 | Dithiosilvatin (**7**) | 0.416 | 0.026 | 0.475 | 0.035 |
| 8 | Aspercolorin (**9**) | 0 | 0 | 0.202 | 0.136 |
| 9 | Aspergillipeptide A (**10**) | 0.609 | 0.008 | 0.722 | 0.009 |
| 10 | Miyakamide A1 (**11**) | 0 | 0 | 0 | 0 |
| 11 | Sulpinine C (**12**) | 0 | 0 | 0.316 | 0.075 |
| 12 | Emericellamide C (**14**) | 0.547 | 0.012 | 0.686 | 0.010 |
| 13 | Ditryptophenaline (**15**) | 0 | 0 | 0 | 0 |
| 14 | Stephacidin B (**16**) | 0 | 0 | 0 | 0 |

‘-’ means not measured.

‘Pa’ assessment of probability for the structure to represent active molecule.

‘Pi’ assessment of probability for the structure to represent inactive molecule.

**Table S3.** AntiBac-pred and AntiFun-pred confidence scores for the selected compounds against *E. coli* and *C. albicans* from PASS online analysis

| Compound | Confidence ratio against *E. coli* | Confidence ratio against *C. albicans* |
| --- | --- | --- |
| Asnipyrone A (**3**) | - | 0 |
| Aspergillipeptide A (**10**) | 0.2391 | 0.0507 |
| Astellatol (**5**) | - | 0.2478 |
| Emericellamide C (**14**) | 0.0937 | 0.1498 |

‘-’ means not measured.

**Table S4.** Results of PharmMapper server for Aspergillipeptide A (**10**) ranked by normalized fit score in descending order.

| **NO.** | **PDB ID** | **Name** | **Number of features** | **Fit score** | **Normalized fit score** |
| --- | --- | --- | --- | --- | --- |
| 1 | 1kev | NADP-dependent alcohol dehydrogenase | 3 | 2.828 | 0.9428 |
| 2 | 3sdl | NONE | 4 | 3.459 | 0.8646 |
| 3 | 1gff | Capsid protein | 3 | 2.5 | 0.8334 |
| 4 | 2rfo | Nucleoporin NIC96 | 4 | 3.043 | 0.7608 |
| 5 | 1uwk | Urocanate hydratase | 4 | 2.987 | 0.7467 |
| 6 | 2cwx | Ribulose bisphosphate carboxylase | 4 | 2.976 | 0.744 |
| 7 | 1a6z | Hereditary hemochromatosis protein | 4 | 2.968 | 0.742 |
| 8 | 1a5t | DNA polymerase III subunit delta | 4 | 2.937 | 0.7342 |
| 9 | 1nsl | Putative ribosomal N-acetyltransferase ydaF | 4 | 2.932 | 0.7329 |
| 10 | 2iwh | Elongation factor 3A | 3 | 2.187 | 0.7291 |
| 11 | 2gnx | UPF0536 protein C12orf66 homolog | 4 | 2.898 | 0.7244 |
| 12 | 1nf0 | Triosephosphate isomerase | 4 | 2.891 | 0.7226 |
| 13 | 1a0i | DNA ligase | 4 | 2.882 | 0.7204 |
| 14 | 2e8x | Geranylgeranyl pyrophosphate synthetase | 4 | 2.879 | 0.7197 |
| 15 | 2rgv | Peroxide operon regulator | 4 | 2.875 | 0.7187 |
| 16 | 1eyb | Homogentisate 1,2-dioxygenase | 4 | 2.868 | 0.717 |
| 17 | 2dbr | Glyoxylate reductase | 4 | 2.864 | 0.7159 |
| 18 | 3fmo | Nuclear pore complex protein Nup214 | 4 | 2.862 | 0.7154 |
| 19 | 2vld | UPF0286 protein PYRAB01260 | 4 | 2.852 | 0.7129 |
| 20 | 1xbw | Heme-degrading monooxygenase isdG | 4 | 2.851 | 0.7127 |
| 21 | 1khv | Genome polyprotein | 4 | 2.83 | 0.7075 |
| 22 | 1gk9 | Penicillin G acylase | 4 | 2.829 | 0.7074 |
| 23 | 1e60 | Dimethyl sulfoxide/trimethylamine N-oxide reductase | 4 | 2.818 | 0.7045 |
| 24 | 1w1g | 3-phosphoinositide-dependent protein kinase 1 | 4 | 2.8 | 0.7 |
| 25 | 1uyr | Acetyl-CoA carboxylase | 4 | 2.8 | 0.6999 |
| 26 | 2ph7 | Uncharacterized protein AF_2093 | 4 | 2.786 | 0.6964 |
| 27 | 1a0j | Trypsin-3 | 4 | 2.759 | 0.6897 |
| 28 | 3cpr | Dihydrodipicolinate synthase | 4 | 2.731 | 0.6827 |
| 29 | 1y8e | Complement control protein | 4 | 2.728 | 0.682 |
| 30 | 1lk3 | Interleukin-10 | 4 | 2.712 | 0.678 |
| 31 | 1e19 | Carbamate kinase | 4 | 2.708 | 0.677 |
| 32 | 1jl2 | Ribonuclease HI | 4 | 2.676 | 0.669 |
| 33 | 2ibg | Protein hedgehog | 5 | 3.298 | 0.6596 |
| 34 | 2veq | Centromere DNA-binding protein complex CBF3 subunit B | 4 | 2.637 | 0.6593 |
| 35 | 1b8h | DNA polymerase processivity component | 4 | 2.619 | 0.6546 |
| 36 | 2cv8 | tRNA-splicing endonuclease | 4 | 2.6 | 0.65 |
| 37 | 1wym | Transgelin-2 | 4 | 2.594 | 0.6485 |
| 38 | 2ebz | Regulator of G-protein signaling 12 | 4 | 2.563 | 0.6406 |
| 39 | 1u94 | Protein recA | 4 | 2.553 | 0.6382 |
| 40 | 3ech | Multidrug resistance operon repressor | 6 | 3.822 | 0.637 |
| 41 | 1jqk | Carbon monoxide dehydrogenase | 4 | 2.535 | 0.6337 |
| 42 | 2nr0 | tRNA pseudouridine synthase A | 4 | 2.529 | 0.6324 |
| 43 | 2co9 | Thymocyte selection-associated high mobility group box protein TOX | 4 | 2.527 | 0.6318 |
| 44 | 1m5y | Chaperone surA | 7 | 4.279 | 0.6113 |
| 45 | 1rso | Disks large homolog 1 | 6 | 3.624 | 0.6039 |
| 46 | 2h9v | Rho-associated protein kinase 2 | 4 | 2.401 | 0.6002 |
| 47 | 1vac | H-2 class I histocompatibility antigen, K-B alpha chain | 5 | 2.993 | 0.5986 |
| 48 | 2z6e | Disheveled-associated activator of morphogenesis 1 | 5 | 2.98 | 0.596 |
| 49 | 1qox | Beta-glucosidase | 5 | 2.979 | 0.5958 |
| 50 | 1ggt | Coagulation factor XIII A chain | 5 | 2.969 | 0.5938 |
| 51 | 1em2 | StAR-related lipid transfer protein 3 | 5 | 2.969 | 0.5938 |
| 52 | 2nz2 | Argininosuccinate synthase | 4 | 2.368 | 0.5921 |
| 53 | 1urj | Major DNA-binding protein | 5 | 2.958 | 0.5916 |
| 54 | 1ycd | Family of serine hydrolases 1 | 5 | 2.949 | 0.5898 |
| 55 | 2i7t | Cleavage and polyadenylation specificity factor subunit 3 | 4 | 2.353 | 0.5884 |
| 56 | 1ovt | Ovotransferrin | 5 | 2.94 | 0.588 |
| 57 | 1ypf | GMP reductase | 5 | 2.925 | 0.585 |
| 58 | 1qca | Chloramphenicol acetyltransferase 3 | 5 | 2.912 | 0.5824 |
| 59 | 3grh | Putative acyl-CoA thioester hydrolase ybhC | 6 | 3.486 | 0.5811 |
| 60 | 2uvn | Putative cytochrome P450 130 | 5 | 2.868 | 0.5737 |
| 61 | 1omw | Beta-adrenergic receptor kinase 1 | 5 | 2.861 | 0.5723 |
| 62 | 2p0r | Calpain-9 | 5 | 2.859 | 0.5719 |
| 63 | 1og4 | T-cell ecto-ADP-ribosyltransferase 2 | 6 | 3.429 | 0.5715 |
| 64 | 1gzq | T-cell surface glycoprotein CD1b | 5 | 2.858 | 0.5715 |
| 65 | 2dfd | Malate dehydrogenase, mitochondrial | 4 | 2.282 | 0.5706 |
| 66 | 1vmk | Purine nucleoside phosphorylase | 5 | 2.852 | 0.5705 |
| 67 | 1p7o | Phospholipase A2, acidic 2 | 6 | 3.422 | 0.5704 |
| 68 | 1njf | DNA polymerase III subunit tau | 6 | 3.415 | 0.5692 |
| 69 | 3exa | tRNA Delta(2)-isopentenylpyrophosphate transferase | 5 | 2.828 | 0.5655 |
| 70 | 1exv | Glycogen phosphorylase, liver form | 5 | 2.825 | 0.5649 |
| 71 | 3dpl | Cullin-5 | 4 | 2.254 | 0.5634 |
| 72 | 1ord | Ornithine decarboxylase, inducible | 5 | 2.817 | 0.5633 |
| 73 | 3eh1 | Protein transport protein Sec24B | 5 | 2.814 | 0.5629 |
| 74 | 1civ | Malate dehydrogenase [NADP], chloroplast | 6 | 3.363 | 0.5605 |
| 75 | 1i3a | Ribonuclease HII | 4 | 2.236 | 0.559 |
| 76 | 1trb | Thioredoxin reductase | 5 | 2.786 | 0.5573 |
| 77 | 1tm9 | Uncharacterized protein MG354 | 5 | 2.785 | 0.557 |
| 78 | 2v62 | Serine/threonine-protein kinase VRK2 | 5 | 2.781 | 0.5562 |
| 79 | 3esw | Peptide-N(4)-(N-acetyl-beta-glucosaminyl)asparagine amidase | 5 | 2.773 | 0.5546 |
| 80 | 1o9k | Transcription factor E2F1 | 5 | 2.77 | 0.5539 |
| 81 | 2da7 | Zinc finger E-box-binding homeobox 2 | 7 | 3.865 | 0.5522 |
| 82 | 3c2o | Nicotinate-nucleotide pyrophosphorylase [carboxylating] | 5 | 2.755 | 0.5509 |
| 83 | 3cmi | Peroxiredoxin HYR1 | 6 | 3.281 | 0.5469 |
| 84 | 1yiv | Myelin P2 protein | 8 | 4.357 | 0.5447 |
| 85 | 1rks | Ribokinase | 5 | 2.72 | 0.5439 |
| 86 | 2cra | Homeobox protein Hox-B13 | 5 | 2.71 | 0.542 |
| 87 | 1qe0 | Histidyl-tRNA synthetase | 6 | 3.226 | 0.5377 |
| 88 | 1cg8 | Hemoglobin subunit alpha | 15 | 8.016 | 0.5344 |
| 89 | 2raj | Sorting nexin-9 | 5 | 2.654 | 0.5307 |
| 90 | 3cm0 | Adenylate kinase | 5 | 2.642 | 0.5284 |
| 91 | 3df0 | Calpain-2 catalytic subunit | 5 | 2.63 | 0.526 |
| 92 | 1wm5 | Neutrophil cytosol factor 2 | 5 | 2.612 | 0.5224 |
| 93 | 2hjs | USG-1 protein homolog | 5 | 2.579 | 0.5158 |
| 94 | 1mwc | Myoglobin | 7 | 3.584 | 0.512 |
| 95 | 1xjv | Protection of telomeres protein 1 | 5 | 2.559 | 0.5118 |
| 96 | 2q5y | Nuclear pore complex protein Nup98-Nup96 | 5 | 2.557 | 0.5114 |
| 97 | 1vrn | Photosynthetic reaction center cytochrome c subunit | 5 | 2.553 | 0.5105 |
| 98 | 2rnx | Histone acetyltransferase KAT2B | 5 | 2.546 | 0.5093 |
| 99 | 1qqj | Fumarylacetoacetase | 6 | 3.055 | 0.5092 |
| 100 | 1bwy | Fatty acid-binding protein, heart | 5 | 2.525 | 0.505 |
| 101 | 2f8n | Histone H3.2 | 5 | 2.522 | 0.5044 |
| 102 | 2ay0 | Bifunctional protein putA | 5 | 2.517 | 0.5035 |
| 103 | 1zq3 | Homeotic protein bicoid | 5 | 2.485 | 0.497 |
| 104 | 1xge | Dihydroorotase | 6 | 2.981 | 0.4968 |
| 105 | 2o9c | Bacteriophytochrome | 8 | 3.967 | 0.4959 |
| 106 | 2z7x | Toll-like receptor 1 | 6 | 2.974 | 0.4957 |
| 107 | 2ccy | Cytochrome c | 6 | 2.974 | 0.4956 |
| 108 | 2gvc | Thiol-specific monooxygenase | 7 | 3.468 | 0.4955 |
| 109 | 2yy8 | tRNA ribose 2-O-methyltransferase aTrm56 | 6 | 2.971 | 0.4951 |
| 110 | 1vrb | Uncharacterized protein yxbC | 6 | 2.968 | 0.4947 |
| 111 | 1to6 | Glycerate kinase | 6 | 2.968 | 0.4946 |
| 112 | 1wf8 | Neurabin-1 | 6 | 2.965 | 0.4942 |
| 113 | 1or7 | RNA polymerase sigma-E factor | 7 | 3.456 | 0.4937 |
| 114 | 1d06 | Sensor protein fixL | 7 | 3.454 | 0.4934 |
| 115 | 3gos | 2,3,4,5-tetrahydropyridine-2,6-dicarboxylate N-succinyltransferase | 6 | 2.961 | 0.4934 |
| 116 | 1txk | Glucans biosynthesis protein G | 6 | 2.957 | 0.4928 |
| 117 | 2yqi | High mobility group protein B3 | 7 | 3.449 | 0.4927 |
| 118 | 1xqv | Proline iminopeptidase | 6 | 2.953 | 0.4922 |
| 119 | 1hj1 | Estrogen receptor beta | 6 | 2.948 | 0.4913 |
| 120 | 2d3w | Probable ATP-dependent transporter sufC | 6 | 2.946 | 0.491 |
| 121 | 1r2j | FkbI | 6 | 2.943 | 0.4904 |
| 122 | 1d8w | L-rhamnose isomerase | 6 | 2.933 | 0.4888 |
| 123 | 1ddm | Protein numb | 5 | 2.442 | 0.4884 |
| 124 | 1tg6 | Putative ATP-dependent Clp protease proteolytic subunit, mitochondrial | 6 | 2.928 | 0.4881 |
| 125 | 1t90 | Methylmalonate semialdehyde dehydrogenase [acylating] | 6 | 2.927 | 0.4878 |
| 126 | 2q3m | Flavonol sulfotransferase-like | 6 | 2.924 | 0.4874 |
| 127 | 1vky | S-adenosylmethionin | 6 | 2.922 | 0.487 |
| 128 | 2dvk | UPF0130 protein APE_0816 | 6 | 2.918 | 0.4864 |
| 129 | 3cb5 | FACT complex subunit spt16 | 6 | 2.914 | 0.4856 |
| 130 | 1dos | Fructose-bisphosphate aldolase class 2 | 6 | 2.911 | 0.4851 |
| 131 | 2gux | Griffithsin | 6 | 2.91 | 0.485 |
| 132 | 1veg | NEDD8 ultimate buster 1 | 6 | 2.91 | 0.485 |
| 133 | 1lnl | Hemocyanin type 2 unit e | 6 | 2.906 | 0.4844 |
| 134 | 3epy | Acyl-CoA-binding domain-containing protein 7 | 6 | 2.905 | 0.4842 |
| 135 | 1tv5 | Dihydroorotate dehydrogenase homolog, mitochondrial | 6 | 2.905 | 0.4841 |
| 136 | 1lrw | Methanol dehydrogenase subunit 1 | 6 | 2.905 | 0.4841 |
| 137 | 1d5b | Ig kappa chain C region | 6 | 2.903 | 0.4838 |
| 138 | 2z3x | Small, acid-soluble spore protein C | 6 | 2.902 | 0.4837 |
| 139 | 2pff | Fatty acid synthase subunit alpha | 6 | 2.886 | 0.481 |
| 140 | 1is2 | Acyl-coenzyme A oxidase 1, peroxisomal | 6 | 2.886 | 0.481 |
| 141 | 1qsa | Soluble lytic murein transglycosylase precursor | 6 | 2.885 | 0.4809 |
| 142 | 3cjh | Mitochondrial import inner membrane translocase subunit TIM13 | 6 | 2.883 | 0.4805 |
| 143 | 1tu9 | Hypothetical protein | 6 | 2.882 | 0.4804 |
| 144 | 3b3d | Uncharacterized oxidoreductase ytbE | 6 | 2.877 | 0.4795 |
| 145 | 2i2l | SPBc2 prophage-derived uncharacterized protein yopX | 6 | 2.872 | 0.4787 |
| 146 | 2zbt | Pyridoxal biosynthesis lyase pdxS | 6 | 2.872 | 0.4787 |
| 147 | 1n46 | Thyroid hormone receptor beta | 6 | 2.872 | 0.4787 |
| 148 | 1vpx | Transaldolase | 6 | 2.867 | 0.4779 |
| 149 | 1fdy | N-acetylneuraminate lyase | 6 | 2.867 | 0.4778 |
| 150 | 3e7w | D-alanine--poly(phosphoribitol) ligase subunit 1 | 6 | 2.865 | 0.4775 |
| 151 | 1xs8 | Probable Fe(2+)-trafficking protein | 6 | 2.864 | 0.4773 |
| 152 | 2qv6 | GTP cyclohydrolase III | 6 | 2.863 | 0.4771 |
| 153 | 3hjb | Glucose-6-phosphate isomerase | 6 | 2.862 | 0.4771 |
| 154 | 1h7y | Translationally controlled tumor protein homolog | 6 | 2.86 | 0.4767 |
| 155 | 1pma | Proteasome subunit alpha | 6 | 2.843 | 0.4738 |
| 156 | 1ukf | Cysteine protease avirulence protein avrPphB | 6 | 2.841 | 0.4735 |
| 157 | 1j7x | Retinol-binding protein 3 | 6 | 2.84 | 0.4734 |
| 158 | 1lns | Xaa-Pro dipeptidyl-peptidase | 5 | 2.363 | 0.4726 |
| 159 | 3c72 | Geranylgeranyl transferase type-2 subunit alpha | 6 | 2.834 | 0.4724 |
| 160 | 1lpv | Protein doublesex | 6 | 2.83 | 0.4717 |
| 161 | 1fsu | Arylsulfatase B | 6 | 2.828 | 0.4713 |
| 162 | 1qgk | Importin subunit beta-1 | 6 | 2.824 | 0.4707 |
| 163 | 1bh9 | Transcription initiation factor TFIID subunit 13 | 6 | 2.816 | 0.4693 |
| 164 | 2fge | Presequence protease 1, chloroplastic/mitochondrial | 6 | 2.811 | 0.4686 |
| 165 | 1jt8 | Translation initiation factor 1A | 6 | 2.809 | 0.4682 |
| 166 | 2uyy | Putative oxidoreductase GLYR1 | 5 | 2.338 | 0.4677 |
| 167 | 1kvd | Salt-mediated killer protoxin 1 | 6 | 2.795 | 0.4658 |
| 168 | 1ma1 | Superoxide dismutase [Fe] | 6 | 2.789 | 0.4649 |
| 169 | 2w9h | Dihydrofolate reductase | 9 | 4.184 | 0.4648 |
| 170 | 2zu6 | Eukaryotic initiation factor 4A-I | 6 | 2.788 | 0.4646 |
| 171 | 2rmr | Paired amphipathic helix protein Sin3a | 6 | 2.787 | 0.4646 |
| 172 | 1w6j | Lanosterol synthase | 6 | 2.775 | 0.4626 |
| 173 | 1xg0 | Phycoerythrin alpha-3 chain, chloroplastic | 6 | 2.763 | 0.4605 |
| 174 | 3ibv | Exportin-T | 6 | 2.749 | 0.4582 |
| 175 | 3eay | Sentrin-specific protease 7 | 7 | 3.201 | 0.4573 |
| 176 | 2hc5 | Uncharacterized protein yvyC | 13 | 5.94 | 0.4569 |
| 177 | 1kvo | Phospholipase A2, membrane associated | 5 | 2.279 | 0.4558 |
| 178 | 1ovm | Indole-3-pyruvate decarboxylase | 6 | 2.734 | 0.4557 |
| 179 | 1imh | Nuclear factor of activated T-cells 5 | 6 | 2.733 | 0.4555 |
| 180 | 2yww | Aspartate carbamoyltransferase regulatory chain | 5 | 2.275 | 0.455 |
| 181 | 2ad1 | Sulfotransferase 1C4 | 6 | 2.725 | 0.4541 |
| 182 | 2z2n | Virginiamycin B lyase | 7 | 3.165 | 0.4522 |
| 183 | 1x9n | DNA ligase 1 | 6 | 2.712 | 0.4519 |
| 184 | 1bsl | Alkanal monooxygenase beta chain | 5 | 2.26 | 0.4519 |
| 185 | 1qwo | 3-phytase A | 6 | 2.707 | 0.4512 |
| 186 | 2d8c | Phosphatidylcholin | 6 | 2.7 | 0.4501 |
| 187 | 1jw2 | Hemolysin expression-modulating protein | 6 | 2.7 | 0.45 |
| 188 | 1we1 | Heme oxygenase 1 | 8 | 3.599 | 0.4499 |
| 189 | 3bom | Hemoglobin subunit alpha-4 | 7 | 3.148 | 0.4497 |
| 190 | 1or6 | Heme-based aerotactic transducer hemAT | 7 | 3.147 | 0.4495 |
| 191 | 3csk | Probable dipeptidyl-peptidase 3 | 6 | 2.693 | 0.4489 |
| 192 | 1s6i | Calcium-dependent protein kinase SK5 | 6 | 2.686 | 0.4477 |
| 193 | 2j4k | Uridylate kinase | 6 | 2.678 | 0.4463 |
| 194 | 2jpe | Nuclear inhibitor of protein phosphatase 1 | 6 | 2.672 | 0.4453 |
| 195 | 2bsk | Mitochondrial import inner membrane translocase subunit Tim9 | 8 | 3.558 | 0.4447 |
| 196 | 3btu | Galactose/lactose metabolism regulatory protein GAL80 | 6 | 2.667 | 0.4445 |
| 197 | 3d98 | Bifunctional protein glmU | 6 | 2.662 | 0.4437 |
| 198 | 1opg | Ig gamma-1 chain C region secreted form | 6 | 2.661 | 0.4434 |
| 199 | 2zat | Dehydrogenase/reductase SDR family member 4 | 6 | 2.66 | 0.4434 |
| 200 | 2f9d | Pre-mRNA branch site protein p14 | 6 | 2.659 | 0.4431 |
| 201 | 2cvo | Probable N-acetyl-gamma-glutamyl-phosphate reductase, chloroplastic | 8 | 3.534 | 0.4417 |
| 202 | 2rkj | Tyrosyl-tRNA synthetase, mitochondrial | 6 | 2.649 | 0.4416 |
| 203 | 2bud | Males-absent on the first protein | 6 | 2.644 | 0.4406 |
| 204 | 3bum | E3 ubiquitin-protein ligase CBL | 6 | 2.642 | 0.4403 |
| 205 | 1q1l | Chorismate synthase | 8 | 3.503 | 0.4379 |
| 206 | 1ipk | Beta-conglycinin, beta chain | 6 | 2.62 | 0.4366 |
| 207 | 1n2c | Nitrogenase molybdenum-iron protein alpha chain | 8 | 3.486 | 0.4357 |
| 208 | 2if2 | Dephospho-CoA kinase | 6 | 2.611 | 0.4352 |
| 209 | 1b7g | Glyceraldehyde-3-phosphate dehydrogenase | 6 | 2.609 | 0.4348 |
| 210 | 1co6 | Cytochrome c2 | 6 | 2.576 | 0.4294 |
| 211 | 2vof | Bcl-2-related protein A1 | 7 | 2.999 | 0.4284 |
| 212 | 1bin | Leghemoglobin A | 7 | 2.993 | 0.4275 |
| 213 | 1mj4 | Sulfite oxidase, mitochondrial | 7 | 2.989 | 0.4269 |
| 214 | 1zno | UPF0244 protein VC_0702 | 8 | 3.404 | 0.4256 |
| 215 | 1w60 | Proliferating cell nuclear antigen | 6 | 2.55 | 0.425 |
| 216 | 1mba | Globin | 7 | 2.969 | 0.4242 |
| 217 | 1nhy | Elongation factor 1-gamma 1 | 7 | 2.968 | 0.4239 |
| 218 | 2pla | Glycerol-3-phosphate dehydrogenase 1-like protein | 6 | 2.542 | 0.4237 |
| 219 | 2qkd | Zinc finger protein ZPR1 | 7 | 2.965 | 0.4236 |
| 220 | 1e29 | Cytochrome c-550 | 7 | 2.953 | 0.4219 |
| 221 | 2a9e | Catalase | 9 | 3.794 | 0.4216 |
| 222 | 2c7y | 3-ketoacyl-CoA thiolase 2, peroxisomal | 7 | 2.947 | 0.4211 |
| 223 | 2h5g | Delta-1-pyrroline-5-carboxylate synthetase | 7 | 2.932 | 0.4189 |
| 224 | 1fok | Type-2 restriction enzyme FokI | 7 | 2.93 | 0.4186 |
| 225 | 1k20 | Probable manganese-dependent inorganic pyrophosphatase | 6 | 2.511 | 0.4185 |
| 226 | 1jh6 | Cyclic phosphodiesterase | 11 | 4.596 | 0.4179 |
| 227 | 1ygh | Histone acetyltransferase GCN5 | 6 | 2.508 | 0.4179 |
| 228 | 2jbp | MAP kinase-activated protein kinase 2 | 8 | 3.34 | 0.4175 |
| 229 | 2r5k | Major capsid protein L1 | 8 | 3.339 | 0.4174 |
| 230 | 3blv | Isocitrate dehydrogenase [NAD] subunit 1, mitochondrial | 9 | 3.756 | 0.4173 |
| 231 | 2e74 | Cytochrome b6 | 7 | 2.921 | 0.4173 |
| 232 | 1ud1 | Glycinin G1 | 7 | 2.92 | 0.4171 |
| 233 | 3cwz | Ras-related protein Rab-6A | 7 | 2.916 | 0.4166 |
| 234 | 2vz6 | Calcium/calmodulin-dependent protein kinase type II alpha chain | 8 | 3.325 | 0.4157 |
| 235 | 1dnv | Coat protein VP1/VP2/VP3/VP4 | 6 | 2.493 | 0.4154 |
| 236 | 2iuk | Seed lipoxygenase | 7 | 2.906 | 0.4152 |
| 237 | 1p4q | Cbp/p300-interacting transactivator 2 | 7 | 2.905 | 0.4151 |
| 238 | 2hh8 | Uncharacterized protein ydfO | 7 | 2.905 | 0.415 |
| 239 | 1ix1 | Peptide deformylase | 7 | 2.904 | 0.4149 |
| 240 | 2agj | Ig mu chain C region | 7 | 2.902 | 0.4146 |
| 241 | 1xpo | Transcription termination factor rho | 7 | 2.898 | 0.4141 |
| 242 | 1b25 | Formaldehyde ferredoxin oxidoreductase | 6 | 2.484 | 0.414 |
| 243 | 2om5 | Contactin-2 | 7 | 2.897 | 0.4139 |
| 244 | 1xdt | Proheparin-binding EGF-like growth factor | 7 | 2.894 | 0.4134 |
| 245 | 2eov | Zinc finger protein 484 | 7 | 2.892 | 0.4131 |
| 246 | 1j3x | High mobility group protein B2 | 7 | 2.89 | 0.4129 |
| 247 | 2oif | Non-symbiotic hemoglobin | 7 | 2.886 | 0.4122 |
| 248 | 2hi7 | Thio | 7 | 2.883 | 0.4119 |
| 249 | 3b46 | Probable kynurenine--oxoglutarate transaminase BNA3 | 8 | 3.295 | 0.4118 |
| 250 | 1o7d | Lysosomal alpha-mannosidase | 7 | 2.883 | 0.4118 |
| 251 | 3egl | UPF0230 protein Cgl2349/cg2579 | 13 | 5.345 | 0.4112 |
| 252 | 2bjh | Feruloyl esterase A | 7 | 2.879 | 0.4112 |
| 253 | 2ju0 | Calcium-binding protein NCS-1 | 7 | 2.879 | 0.4112 |
| 254 | 1skq | Elongation factor 1-alpha | 8 | 3.287 | 0.4109 |
| 255 | 1v8b | Adenosylhomocysteinase | 7 | 2.875 | 0.4107 |
| 256 | 2rmp | Mucorpepsin | 7 | 2.865 | 0.4092 |
| 257 | 1miu | Breast cancer type 2 susceptibility protein homolog | 8 | 3.273 | 0.4091 |
| 258 | 1we5 | Alpha-xylosidase | 7 | 2.861 | 0.4087 |
| 259 | 3e7g | Nitric oxide synthase, inducible | 7 | 2.859 | 0.4084 |
| 260 | 1bq6 | Chalcone synthase 2 | 7 | 2.854 | 0.4077 |
| 261 | 1tvm | Galactitol-specific phosphotransferase enzyme IIB component | 7 | 2.854 | 0.4077 |
| 262 | 2ex8 | D-alanyl-D-alanine carboxypeptidase dacB | 6 | 2.439 | 0.4065 |
| 263 | 3f9i | 3-oxoacyl-[acyl-carrier-protein] reductase | 7 | 2.843 | 0.4062 |
| 264 | 1wgw | Signal recognition particle 54 kDa protein | 7 | 2.84 | 0.4057 |
| 265 | 2p2c | Caspase-2 | 7 | 2.84 | 0.4057 |
| 266 | 2v4j | Sulfite reductase, dissimilatory-type subunit alpha | 7 | 2.833 | 0.4047 |
| 267 | 1qib | 72 kDa type IV collagenase | 8 | 3.236 | 0.4044 |
| 268 | 2e5o | Activating signal cointegrator 1 | 6 | 2.426 | 0.4043 |
| 269 | 2pnc | Primary amine oxidase, liver isozyme | 7 | 2.829 | 0.4042 |
| 270 | 1prx | Peroxiredoxin-6 | 7 | 2.828 | 0.404 |
| 271 | 2cr7 | Paired amphipathic helix protein Sin3b | 7 | 2.827 | 0.4038 |
| 272 | 1mc8 | Flap structure-specific endonuclease | 7 | 2.824 | 0.4034 |
| 273 | 1yf6 | Reaction center protein H chain | 7 | 2.823 | 0.4033 |
| 274 | 2vv5 | Small-conductance mechanosensitive channel | 7 | 2.823 | 0.4032 |
| 275 | 1w3b | UDP-N-acetylglucosamine--peptide N-acetylglucosaminyltransferase 110 kDa subunit | 7 | 2.822 | 0.4031 |
| 276 | 1vr3 | 1,2-dihydroxy-3-keto-5-methylthiopentene dioxygenase | 7 | 2.82 | 0.4028 |
| 277 | 1hn0 | Chondroitin ABC endolyase 1 | 6 | 2.415 | 0.4025 |
| 278 | 1gv0 | Malate dehydrogenase | 9 | 3.621 | 0.4023 |
| 279 | 2qq5 | Dehydrogenase/reductase SDR family member 1 | 7 | 2.813 | 0.4019 |
| 280 | 1nj8 | Prolyl-tRNA synthetase | 7 | 2.808 | 0.4011 |
| 281 | 3dwl | Actin-related protein 3 | 7 | 2.805 | 0.4007 |
| 282 | 1n97 | Cytochrome P450 | 9 | 3.604 | 0.4005 |
| 283 | 2nzt | Hexokinase-2 | 7 | 2.804 | 0.4005 |
| 284 | 1n8w | Malate synthase G | 7 | 2.799 | 0.3999 |
| 285 | 1d5y | Right origin-binding protein | 8 | 3.195 | 0.3994 |
| 286 | 1c0l | D-amino-acid oxidase | 7 | 2.788 | 0.3983 |
| 287 | 2h2u | Soluble calcium-activated nucleotidase 1 | 7 | 2.788 | 0.3983 |
| 288 | 2hzt | Uncharacterized HTH-type transcriptional regulator ytcD | 7 | 2.785 | 0.3979 |
| 289 | 1x7z | 2-oxoisovalerate dehydrogenase subunit alpha, mitochondrial | 6 | 2.385 | 0.3975 |
| 290 | 1fe8 | von Willebrand factor | 6 | 2.38 | 0.3967 |
| 291 | 2qc7 | Endoplasmic reticulum protein ERp29 | 7 | 2.775 | 0.3965 |
| 292 | 2qnc | Recombination endonuclease VII | 7 | 2.774 | 0.3963 |
| 293 | 2dtg | Insulin receptor | 7 | 2.772 | 0.396 |
| 294 | 2akj | Ferredoxin--nitrite reductase, chloroplastic | 7 | 2.771 | 0.3959 |
| 295 | 2nro | Molybdopterin biosynthesis protein moeA | 8 | 3.164 | 0.3956 |
| 296 | 1orr | CDP-paratose 2-epimerase | 7 | 2.765 | 0.395 |
| 297 | 2dfj | Bis(5-nucleosyl)-tetraphosphatase, symmetrical | 7 | 2.763 | 0.3948 |
| 298 | 1h9u | Retinoic acid receptor RXR-beta | 6 | 2.369 | 0.3948 |
| 299 | 1ouu | Hemoglobin subunit alpha-1 | 9 | 3.547 | 0.3941 |

**Table S5.** Results of PharmMapper server for Emericellamide C (**14**) ranked by normalized fit score in descending order.

| No. | PDB ID | Name | **Number of features** | **Fit Score** | **Normalized Fit Score** |
| --- | --- | --- | --- | --- | --- |
| 1 | 1kev | NADP-dependent alcohol dehydrogenase | 3 | 2.655 | 0.8851 |
| 2 | 1gff | Capsid protein | 3 | 2.626 | 0.8753 |
| 3 | 1ojm | Hyaluronate lyase | 4 | 2.992 | 0.748 |
| 4 | 1rk5 | NONE | 4 | 2.974 | 0.7434 |
| 5 | 1wfh | Zinc finger A20 and AN1 domain-containing stress-associated protein 4 | 4 | 2.962 | 0.7405 |
| 6 | 1q8c | Uncharacterized protein MG027 | 4 | 2.96 | 0.7401 |
| 7 | 9aat | Aspartate aminotransferase, mitochondrial | 4 | 2.94 | 0.7349 |
| 8 | 1jqk | Carbon monoxide dehydrogenase | 4 | 2.93 | 0.7325 |
| 9 | 1ig8 | Hexokinase-2 | 4 | 2.919 | 0.7299 |
| 10 | 2cop | Acyl-CoA-binding domain-containing protein 6 | 4 | 2.913 | 0.7283 |
| 11 | 1nvm | 4-hydroxy-2-oxovalerate aldolase | 4 | 2.91 | 0.7276 |
| 12 | 1u94 | Protein recA | 4 | 2.91 | 0.7274 |
| 13 | 2ph7 | Uncharacterized protein AF_2093 | 4 | 2.885 | 0.7213 |
| 14 | 2aeu | UPF0425 pyridoxal phosphate-dependent protein MJ0158 | 4 | 2.875 | 0.7187 |
| 15 | 2rfo | Nucleoporin NIC96 | 4 | 2.873 | 0.7184 |
| 16 | 2yrv | AT-rich interactive domain-containing protein 4A | 4 | 2.867 | 0.7168 |
| 17 | 1w1g | 3-phosphoinositide-dependent protein kinase 1 | 4 | 2.862 | 0.7154 |
| 18 | 1irz | Two-component response regulator ARR10 | 4 | 2.85 | 0.7126 |
| 19 | 3cpr | Dihydrodipicolinate synthase | 4 | 2.849 | 0.7122 |
| 20 | 2dfd | Malate dehydrogenase, mitochondrial | 4 | 2.842 | 0.7106 |
| 21 | 1d2e | Elongation factor Tu, mitochondrial | 4 | 2.837 | 0.7094 |
| 22 | 2dba | Protein unc-45 homolog A | 4 | 2.83 | 0.7075 |
| 23 | 3fmo | Nuclear pore complex protein Nup214 | 4 | 2.82 | 0.7049 |
| 24 | 1e19 | Carbamate kinase | 4 | 2.816 | 0.704 |
| 25 | 2hma | tRNA-specific 2-thiouridylase mnmA | 4 | 2.809 | 0.7023 |
| 26 | 2i7x | Cleavage and polyadenylation specificity factor subunit 3 | 4 | 2.805 | 0.7011 |
| 27 | 1k99 | Nucleolar transcription factor 1 | 4 | 2.802 | 0.7005 |
| 28 | 2ig7 | Choline/ethanolamine kinase | 4 | 2.796 | 0.6991 |
| 29 | 3dpl | Cullin-5 | 4 | 2.79 | 0.6976 |
| 30 | 3bx1 | Subtilisin Savinase | 4 | 2.788 | 0.697 |
| 31 | 1p9o | Phosphopantothenate--cysteine ligase | 4 | 2.781 | 0.6953 |
| 32 | 1khv | Genome polyprotein | 4 | 2.77 | 0.6924 |
| 33 | 1sgf | Kallikrein 1-related peptidase-like b4 | 4 | 2.743 | 0.6858 |
| 34 | 1m1j | Fibrinogen alpha chain | 4 | 2.736 | 0.6839 |
| 35 | 3fer | Filamin-B | 4 | 2.724 | 0.681 |
| 36 | 2cv8 | tRNA-splicing endonuclease | 4 | 2.711 | 0.6776 |
| 37 | 1m41 | Alkanesulfonate monooxygenase | 4 | 2.697 | 0.6743 |
| 38 | 1wym | Transgelin-2 | 4 | 2.685 | 0.6712 |
| 39 | 1k3j | Serine/threonine-protein kinase RAD53 | 4 | 2.671 | 0.6676 |
| 40 | 3cki | ADAM 17 | 4 | 2.663 | 0.6657 |
| 41 | 1s40 | Cell division control protein 13 | 6 | 3.992 | 0.6653 |
| 42 | 2nz2 | Argininosuccinate synthase | 4 | 2.636 | 0.659 |
| 43 | 2e8x | Geranylgeranyl pyrophosphate synthetase | 4 | 2.634 | 0.6584 |
| 44 | 2a90 | Protein deltex | 4 | 2.623 | 0.6558 |
| 45 | 2pjw | Class E vacuolar protein-sorting machinery protein HSE1 | 4 | 2.622 | 0.6554 |
| 46 | 2gsy | Structural polyprotein | 4 | 2.62 | 0.6551 |
| 47 | 1hm9 | Bifunctional protein glmU | 5 | 3.174 | 0.6349 |
| 48 | 2ibg | Protein hedgehog | 5 | 3.103 | 0.6206 |
| 49 | 1wde | Probable diphthine synthase | 5 | 3.037 | 0.6075 |
| 50 | 3d3l | Arachidonate 12-lipoxygenase, 12S-type | 5 | 3 | 0.6 |
| 51 | 1cg8 | Hemoglobin subunit alpha | 15 | 8.949 | 0.5966 |
| 52 | 1gm6 | Salivary lipocalin | 5 | 2.968 | 0.5937 |
| 53 | 2cra | Homeobox protein Hox-B13 | 5 | 2.959 | 0.5919 |
| 54 | 1wm5 | Neutrophil cytosol factor 2 | 5 | 2.943 | 0.5886 |
| 55 | 2btv | Core protein VP3 | 5 | 2.939 | 0.5877 |
| 56 | 1nu9 | Prothrombin | 5 | 2.935 | 0.5871 |
| 57 | 1tjl | DnaK suppressor protein | 5 | 2.925 | 0.585 |
| 58 | 1kvo | Phospholipase A2, membrane associated | 5 | 2.924 | 0.5848 |
| 59 | 1omw | Beta-adrenergic receptor kinase 1 | 5 | 2.916 | 0.5833 |
| 60 | 2ayn | Ubiquitin carboxyl-terminal hydrolase 14 | 5 | 2.916 | 0.5832 |
| 61 | 1n35 | RNA-directed RNA polymerase lambda-3 | 5 | 2.911 | 0.5822 |
| 62 | 1mg6 | Phospholipase A2 homolog acutohaemolysin | 5 | 2.903 | 0.5805 |
| 63 | 3eh1 | Protein transport protein Sec24B | 5 | 2.888 | 0.5777 |
| 64 | 2hc5 | Uncharacterized protein yvyC | 13 | 7.493 | 0.5764 |
| 65 | 2qpo | Thymidine kinase | 5 | 2.874 | 0.5748 |
| 66 | 2q5y | Nuclear pore complex protein Nup98-Nup96 | 5 | 2.872 | 0.5744 |
| 67 | 1cpc | C-phycocyanin-1 alpha chain | 5 | 2.871 | 0.5743 |
| 68 | 3dm0 | Guanine nucleotide-binding protein subunit beta-like protein | 5 | 2.867 | 0.5734 |
| 69 | 2vig | Short-chain specific acyl-CoA dehydrogenase, mitochondrial | 5 | 2.863 | 0.5726 |
| 70 | 1y1u | Signal transducer and activator of transcription 5A | 5 | 2.859 | 0.5718 |
| 71 | 2yww | Aspartate carbamoyltransferase regulatory chain | 5 | 2.845 | 0.569 |
| 72 | 1bwy | Fatty acid-binding protein, heart | 5 | 2.832 | 0.5664 |
| 73 | 1vjh | Uncharacterized protein At1g24000 | 5 | 2.828 | 0.5656 |
| 74 | 2h9v | Rho-associated protein kinase 2 | 5 | 2.824 | 0.5649 |
| 75 | 1buc | Acyl-CoA dehydrogenase, short-chain specific | 5 | 2.823 | 0.5647 |
| 76 | 1lns | Xaa-Pro dipeptidyl-peptidase | 5 | 2.821 | 0.5642 |
| 77 | 3c2o | Nicotinate-nucleotide pyrophosphorylase [carboxylating] | 5 | 2.818 | 0.5635 |
| 78 | 1b7a | Phosphatidylethanolamine-binding protein 1 | 5 | 2.806 | 0.5611 |
| 79 | 1qib | 72 kDa type IV collagenase | 8 | 4.477 | 0.5596 |
| 80 | 1vae | Rhophilin-2 | 5 | 2.79 | 0.558 |
| 81 | 1djh | 1-phosphatidylinositol-4,5-bisphosphate phosphodiesterase delta-1 | 5 | 2.785 | 0.557 |
| 82 | 1yxo | 4-hydroxythreonine-4-phosphate dehydrogenase 1 | 6 | 3.341 | 0.5568 |
| 83 | 2vpk | Myoneurin | 5 | 2.779 | 0.5558 |
| 84 | 3df0 | Calpain-2 catalytic subunit | 5 | 2.776 | 0.5552 |
| 85 | 1ypf | GMP reductase | 5 | 2.773 | 0.5545 |
| 86 | 2dgu | Heterogeneous nuclear ribonucleoprotein Q | 5 | 2.77 | 0.554 |
| 87 | 1lbm | N-(5-phosphoribosyl)anthranilate isomerase | 5 | 2.768 | 0.5536 |
| 88 | 1fcx | Retinoic acid receptor gamma | 5 | 2.767 | 0.5534 |
| 89 | 2b3y | Cytoplasmic aconitate hydratase | 5 | 2.746 | 0.5492 |
| 90 | 1yel | B3 domain-containing protein At1g16640 | 5 | 2.742 | 0.5484 |
| 91 | 2iu3 | Bifunctional purine biosynthesis protein PURH | 5 | 2.739 | 0.5477 |
| 92 | 3gh8 | Iodotyrosine dehalogenase 1 | 5 | 2.726 | 0.5451 |
| 93 | 2g18 | Phycocyanobili | 5 | 2.71 | 0.542 |
| 94 | 2fv4 | Kinetochore protein SPC25 | 5 | 2.71 | 0.5419 |
| 95 | 1ukf | Cysteine protease avirulence protein avrPphB | 6 | 3.243 | 0.5406 |
| 96 | 2awa | DNA polymerase III subunit beta | 5 | 2.699 | 0.5397 |
| 97 | 2p0r | Calpain-9 | 5 | 2.695 | 0.539 |
| 98 | 1go2 | Ferredoxin--NADP reductase | 5 | 2.685 | 0.537 |
| 99 | 2e5o | Activating signal cointegrator 1 | 6 | 3.222 | 0.5369 |
| 100 | 2nq5 | 5-methyltetrahydropteroyltriglutamate--homocysteine methyltransferase | 5 | 2.682 | 0.5365 |
| 101 | 2c0p | Acetylcholinesterase | 5 | 2.651 | 0.5303 |
| 102 | 1dus | Protein MJ0882 | 6 | 3.17 | 0.5283 |
| 103 | 2bz0 | GTP cyclohydrolase-2 | 6 | 3.166 | 0.5277 |
| 104 | 1odh | Chorion-specific transcription factor GCMa | 5 | 2.634 | 0.5267 |
| 105 | 1lsh | Vitellogenin | 5 | 2.629 | 0.5257 |
| 106 | 1y64 | Actin, alpha skeletal muscle | 8 | 4.183 | 0.5229 |
| 107 | 1yiv | Myelin P2 protein | 8 | 4.094 | 0.5117 |
| 108 | 3bix | Neuroligin-1 | 6 | 3.05 | 0.5084 |
| 109 | 2z6c | Phototropin-1 | 7 | 3.511 | 0.5015 |
| 110 | 2h08 | Ribose-phosphate pyrophosphokinase 1 | 6 | 3.007 | 0.5012 |
| 111 | 1w60 | Proliferating cell nuclear antigen | 6 | 3.006 | 0.5011 |
| 112 | 1njk | Long-chain acyl-CoA thioesterase tesC | 6 | 2.992 | 0.4987 |
| 113 | 2dnk | CUG-BP- and ETR-3-like factor 4 | 6 | 2.992 | 0.4986 |
| 114 | 1hyh | L-2-hydroxyisocaproate dehydrogenase | 6 | 2.968 | 0.4947 |
| 115 | 1qe0 | Histidyl-tRNA synthetase | 6 | 2.961 | 0.4936 |
| 116 | 1kmh | ATP synthase subunit alpha, chloroplastic | 6 | 2.961 | 0.4934 |
| 117 | 2ceo | Thyroxine-binding globulin | 6 | 2.958 | 0.493 |
| 118 | 1o4s | Aspartate aminotransferase | 6 | 2.957 | 0.4929 |
| 119 | 1igw | Isocitrate lyase | 6 | 2.953 | 0.4921 |
| 120 | 1h4j | Methanol dehydrogenase subunit 1 | 6 | 2.948 | 0.4913 |
| 121 | 1hbp | Retinol-binding protein 4 | 6 | 2.946 | 0.4909 |
| 122 | 2i5p | Glyceraldehyde-3-phosphate dehydrogenase 1 | 6 | 2.939 | 0.4899 |
| 123 | 2kdf | 26S proteasome non-ATPase regulatory subunit 4 | 6 | 2.939 | 0.4898 |
| 124 | 1opg | Ig gamma-1 chain C region secreted form | 6 | 2.939 | 0.4898 |
| 125 | 1yf6 | Reaction center protein H chain | 6 | 2.938 | 0.4897 |
| 126 | 2dvk | UPF0130 protein APE_0816 | 6 | 2.936 | 0.4894 |
| 127 | 1lo1 | Steroid hormone receptor ERR2 | 6 | 2.935 | 0.4892 |
| 128 | 1yt5 | Probable inorganic polyphosphate/ATP-NAD kinase | 6 | 2.935 | 0.4891 |
| 129 | 3dpi | NH(3)-dependent NAD(+) synthetase | 6 | 2.929 | 0.4881 |
| 130 | 1xpo | Transcription termination factor rho | 7 | 3.412 | 0.4875 |
| 131 | 1fe8 | von Willebrand factor | 6 | 2.925 | 0.4875 |
| 132 | 1qsa | Soluble lytic murein transglycosylase precursor | 6 | 2.92 | 0.4867 |
| 133 | 1m5s | Formylmethanofuran--tetrahydromethanopterin formyltransferase | 6 | 2.92 | 0.4867 |
| 134 | 2jqq | Conserved oligomeric Golgi complex subunit 2 | 6 | 2.919 | 0.4865 |
| 135 | 3cl5 | Hemagglutinin-esterase | 6 | 2.917 | 0.4862 |
| 136 | 3fqd | 5-3 exoribonuclease 2 | 6 | 2.917 | 0.4862 |
| 137 | 1vrb | Uncharacterized protein yxbC | 6 | 2.909 | 0.4849 |
| 138 | 1gq2 | NADP-dependent malic enzyme | 6 | 2.909 | 0.4848 |
| 139 | 1i4n | Indole-3-glycerol phosphate synthase | 6 | 2.906 | 0.4844 |
| 140 | 2gb5 | NADH pyrophosphatase | 6 | 2.906 | 0.4843 |
| 141 | 2k2q | Tyrocidine synthetase 3 | 6 | 2.904 | 0.484 |
| 142 | 2fiq | D-tagatose-1,6-bisphosphate aldolase subunit gatZ | 6 | 2.903 | 0.4838 |
| 143 | 1xs8 | Probable Fe(2+)-trafficking protein | 6 | 2.902 | 0.4837 |
| 144 | 1yy3 | S-adenosylmethionin | 6 | 2.901 | 0.4834 |
| 145 | 3cdk | Probable succinyl-Co | 6 | 2.9 | 0.4833 |
| 146 | 1chm | Creatinase | 6 | 2.897 | 0.4828 |
| 147 | 3csk | Probable dipeptidyl-peptidase 3 | 6 | 2.897 | 0.4828 |
| 148 | 2a2d | N-acetylgalactosamine kinase | 6 | 2.889 | 0.4815 |
| 149 | 1is2 | Acyl-coenzyme A oxidase 1, peroxisomal | 6 | 2.889 | 0.4814 |
| 150 | 2egw | Ribosomal RNA small subunit methyltransferase E | 6 | 2.888 | 0.4814 |
| 151 | 1wf8 | Neurabin-1 | 6 | 2.883 | 0.4805 |
| 152 | 2a5s | Glutamate [NMDA] receptor subunit epsilon-1 | 6 | 2.882 | 0.4803 |
| 153 | 2i0f | 6,7-dimethyl-8-ribityllumazine synthase 1 | 6 | 2.877 | 0.4795 |
| 154 | 2ac0 | Cellular tumor antigen p53 | 6 | 2.876 | 0.4793 |
| 155 | 2q2e | Type II DNA topoisomerase VI subunit A | 6 | 2.874 | 0.479 |
| 156 | 2ols | Phosphoenolpyruvate synthase | 6 | 2.872 | 0.4786 |
| 157 | 2qxx | Deoxycytidine triphosphate deaminase | 8 | 3.828 | 0.4785 |
| 158 | 1zp2 | RNA polymerase II holoenzyme cyclin-like subunit | 6 | 2.867 | 0.4778 |
| 159 | 3cgu | Protein giant-lens | 6 | 2.867 | 0.4778 |
| 160 | 2bjh | Feruloyl esterase A | 7 | 3.344 | 0.4777 |
| 161 | 3dax | Cytochrome P450 7A1 | 6 | 2.866 | 0.4777 |
| 162 | 3hjb | Glucose-6-phosphate isomerase | 6 | 2.866 | 0.4776 |
| 163 | 2b9h | Mitogen-activated protein kinase FUS3 | 7 | 3.341 | 0.4773 |
| 164 | 1k3v | Coat protein VP1 | 6 | 2.861 | 0.4769 |
| 165 | 3cmi | Peroxiredoxin HYR1 | 6 | 2.86 | 0.4767 |
| 166 | 1b25 | Formaldehyde ferredoxin oxidoreductase | 6 | 2.86 | 0.4767 |
| 167 | 2au3 | DNA primase | 6 | 2.857 | 0.4761 |
| 168 | 2uzf | Naphthoate synthase | 6 | 2.855 | 0.4759 |
| 169 | 1t3b | Thio | 6 | 2.855 | 0.4758 |
| 170 | 1f7s | Actin-depolymerizing factor 1 | 6 | 2.855 | 0.4758 |
| 171 | 1nt2 | Fibrillarin-like rRNA/tRNA 2-O-methyltransferase | 6 | 2.855 | 0.4758 |
| 172 | 1xge | Dihydroorotase | 6 | 2.854 | 0.4757 |
| 173 | 1s0w | Beta-lactamase TEM | 6 | 2.852 | 0.4754 |
| 174 | 3c7g | Arabinoxylan arabinofuranohydrolase | 6 | 2.849 | 0.4748 |
| 175 | 1v1f | Calcineurin B-like protein 4 | 6 | 2.849 | 0.4748 |
| 176 | 2gov | Heme-binding protein 1 | 6 | 2.848 | 0.4746 |
| 177 | 1f6t | Nucleoside diphosphate kinase, cytosolic | 8 | 3.796 | 0.4744 |
| 178 | 1tv5 | Dihydroorotate dehydrogenase homolog, mitochondrial | 6 | 2.846 | 0.4743 |
| 179 | 1kya | Laccase-1 | 6 | 2.844 | 0.474 |
| 180 | 2pff | Fatty acid synthase subunit alpha | 6 | 2.834 | 0.4724 |
| 181 | 1yy7 | Regulator of transcription; stringent starvation protein A | 6 | 2.834 | 0.4724 |
| 182 | 1p4t | Surface protein A | 6 | 2.833 | 0.4722 |
| 183 | 1d8w | L-rhamnose isomerase | 6 | 2.832 | 0.472 |
| 184 | 2pla | Glycerol-3-phosphate dehydrogenase 1-like protein | 6 | 2.832 | 0.472 |
| 185 | 2zxh | tRNA uridine 5-carboxymethylaminomethyl modification enzyme mnmG | 6 | 2.83 | 0.4717 |
| 186 | 2hzv | Nickel-responsive regulator | 7 | 3.302 | 0.4716 |
| 187 | 2uxw | Very long-chain specific acyl-CoA dehydrogenase, mitochondrial | 6 | 2.83 | 0.4716 |
| 188 | 2o9c | Bacteriophytochrome | 8 | 3.771 | 0.4714 |
| 189 | 1oyz | Protein yibA | 6 | 2.828 | 0.4713 |
| 190 | 2d3w | Probable ATP-dependent transporter sufC | 6 | 2.827 | 0.4711 |
| 191 | 2spo | Myoglobin | 6 | 2.826 | 0.4711 |
| 192 | 2a8e | UPF0637 protein yktB | 6 | 2.826 | 0.471 |
| 193 | 2lh5 | Leghemoglobin-2 | 8 | 3.767 | 0.4709 |
| 194 | 2fxt | Mitochondrial import inner membrane translocase subunit TIM44 | 6 | 2.826 | 0.4709 |
| 195 | 1l3g | Transcription factor MBP1 | 6 | 2.824 | 0.4707 |
| 196 | 2nn6 | Exosome complex exonuclease RRP45 | 6 | 2.824 | 0.4706 |
| 197 | 2pa6 | Enolase | 6 | 2.822 | 0.4703 |
| 198 | 1vm6 | Dihydrodipicolinate reductase | 6 | 2.817 | 0.4694 |
| 199 | 1x5n | Harmonin | 6 | 2.811 | 0.4686 |
| 200 | 1k20 | Probable manganese-dependent inorganic pyrophosphatase | 6 | 2.811 | 0.4685 |
| 201 | 1fiz | Acrosin | 6 | 2.807 | 0.4679 |
| 202 | 1s70 | Serine/threonine-protein phosphatase PP1-beta catalytic subunit | 6 | 2.807 | 0.4678 |
| 203 | 1in7 | Holliday junction ATP-dependent DNA helicase ruvB | 6 | 2.805 | 0.4676 |
| 204 | 2qv6 | GTP cyclohydrolase III | 6 | 2.802 | 0.4671 |
| 205 | 2ph4 | Phospholipase A2 homolog zhaoermiatoxin | 7 | 3.268 | 0.4669 |
| 206 | 3bua | Telomeric repeat-binding factor 2 | 6 | 2.791 | 0.4652 |
| 207 | 1w98 | G1/S-specific cyclin-E1 | 6 | 2.791 | 0.4651 |
| 208 | 1x9n | DNA ligase 1 | 6 | 2.783 | 0.4639 |
| 209 | 1nfh | DNA/RNA-binding protein Alba 2 | 6 | 2.779 | 0.4632 |
| 210 | 2z3x | Small, acid-soluble spore protein C | 6 | 2.777 | 0.4628 |
| 211 | 1gup | Galactose-1-phosphate uridylyltransferase | 6 | 2.776 | 0.4627 |
| 212 | 1kyi | ATP-dependent hsl protease ATP-binding subunit hslU | 6 | 2.773 | 0.4622 |
| 213 | 3bkb | Proto-oncogene tyrosine-protein kinase Fes/Fps | 6 | 2.773 | 0.4621 |
| 214 | 1qgd | Transketolase 1 | 6 | 2.771 | 0.4618 |
| 215 | 1duw | Nonaheme cytochrome c | 6 | 2.77 | 0.4616 |
| 216 | 2ikq | Ubiquitin-associated and SH3 domain-containing protein B | 6 | 2.77 | 0.4616 |
| 217 | 2i2l | SPBc2 prophage-derived uncharacterized protein yopX | 6 | 2.77 | 0.4616 |
| 218 | 1ws1 | Peptide deformylase 1 | 6 | 2.768 | 0.4614 |
| 219 | 1wh0 | Ubiquitin carboxyl-terminal hydrolase 19 | 6 | 2.768 | 0.4613 |
| 220 | 1x7z | 2-oxoisovalerate dehydrogenase subunit alpha, mitochondrial | 6 | 2.766 | 0.461 |
| 221 | 2bwn | 5-aminolevulinate synthase | 6 | 2.762 | 0.4603 |
| 222 | 1kvd | Salt-mediated killer protoxin 1 | 6 | 2.76 | 0.46 |
| 223 | 1idj | Pectin lyase A | 6 | 2.756 | 0.4593 |
| 224 | 1d7c | Cellobiose dehydrogenase | 6 | 2.754 | 0.4591 |
| 225 | 1r2j | FkbI | 6 | 2.749 | 0.4582 |
| 226 | 1fml | Retinol dehydratase | 6 | 2.749 | 0.4581 |
| 227 | 1njf | DNA polymerase III subunit tau | 6 | 2.748 | 0.458 |
| 228 | 2o7c | Methionine gamma-lyase | 6 | 2.748 | 0.4579 |
| 229 | 1civ | Malate dehydrogenase [NADP], chloroplast | 6 | 2.747 | 0.4578 |
| 230 | 1og4 | T-cell ecto-ADP-ribosyltransferase 2 | 6 | 2.747 | 0.4578 |
| 231 | 1rqg | Methionyl-tRNA synthetase | 6 | 2.746 | 0.4576 |
| 232 | 2zat | Dehydrogenase/reductase SDR family member 4 | 6 | 2.744 | 0.4573 |
| 233 | 2ad1 | Sulfotransferase 1C4 | 6 | 2.743 | 0.4572 |
| 234 | 1fok | Type-2 restriction enzyme FokI | 7 | 3.198 | 0.4569 |
| 235 | 3cj9 | Ectonucleoside triphosphate diphosphohydrolase 2 | 6 | 2.733 | 0.4555 |
| 236 | 1wuu | Galactokinase | 6 | 2.726 | 0.4544 |
| 237 | 2yy8 | tRNA ribose 2-O-methyltransferase aTrm56 | 6 | 2.719 | 0.4532 |
| 238 | 2js1 | Uncharacterized protein yvfG | 6 | 2.713 | 0.4522 |
| 239 | 1j5s | Uronate isomerase | 6 | 2.712 | 0.4521 |
| 240 | 1k39 | 3,2-trans-enoyl-CoA isomerase | 6 | 2.71 | 0.4516 |
| 241 | 1s6b | Phospholipase A2 isoform 1 | 6 | 2.709 | 0.4515 |
| 242 | 2if2 | Dephospho-CoA kinase | 6 | 2.707 | 0.4511 |
| 243 | 2d9n | Cleavage and polyadenylation specificity factor subunit 4 | 6 | 2.707 | 0.4511 |
| 244 | 3c72 | Geranylgeranyl transferase type-2 subunit alpha | 6 | 2.705 | 0.4509 |
| 245 | 2vde | Outer membrane protein tolC | 6 | 2.697 | 0.4495 |
| 246 | 1rso | Disks large homolog 1 | 6 | 2.691 | 0.4485 |
| 247 | 1lnl | Hemocyanin type 2 unit e | 6 | 2.689 | 0.4482 |
| 248 | 2p39 | Fibroblast growth factor 23 | 6 | 2.684 | 0.4474 |
| 249 | 1ign | DNA-binding protein RAP1 | 24 | 10.73 | 0.4472 |
| 250 | 2f4m | Peptide-N(4)-(N-acetyl-beta-glucosaminyl)asparagine amidase | 6 | 2.682 | 0.4469 |
| 251 | 1txk | Glucans biosynthesis protein G | 6 | 2.678 | 0.4463 |
| 252 | 3egl | UPF0230 protein Cgl2349/cg2579 | 13 | 5.786 | 0.4451 |
| 253 | 2ex8 | D-alanyl-D-alanine carboxypeptidase dacB | 6 | 2.662 | 0.4437 |
| 254 | 2bsk | Mitochondrial import inner membrane translocase subunit Tim9 | 8 | 3.542 | 0.4427 |
| 255 | 3ee7 | Replicase polyprotein 1a | 6 | 2.643 | 0.4405 |
| 256 | 1ma1 | Superoxide dismutase [Fe] | 6 | 2.642 | 0.4404 |
| 257 | 1ord | Ornithine decarboxylase, inducible | 10 | 4.398 | 0.4398 |
| 258 | 2yu4 | E3 SUMO-protein ligase NSE2 | 6 | 2.638 | 0.4397 |
| 259 | 1vp2 | Nucleoside-triphosphatase | 9 | 3.956 | 0.4395 |
| 260 | 1kgd | Peripheral plasma membrane protein CASK | 6 | 2.636 | 0.4394 |
| 261 | 1vhn | Hypothetical protein | 6 | 2.636 | 0.4393 |
| 262 | 1pma | Proteasome subunit alpha | 6 | 2.635 | 0.4392 |
| 263 | 1d5b | Ig kappa chain C region | 6 | 2.628 | 0.438 |
| 264 | 2z7x | Toll-like receptor 1 | 6 | 2.627 | 0.4378 |
| 265 | 3grh | Putative acyl-CoA thioester hydrolase ybhC | 6 | 2.625 | 0.4375 |
| 266 | 3cuo | Probable HTH-type transcriptional regulator ygaV | 6 | 2.624 | 0.4374 |
| 267 | 3prc | Photosynthetic reaction center cytochrome c subunit | 7 | 3.059 | 0.437 |
| 268 | 1we1 | Heme oxygenase 1 | 8 | 3.493 | 0.4366 |
| 269 | 2qc3 | Malonyl CoA-acyl carrier protein transacylase | 9 | 3.907 | 0.4342 |
| 270 | 1wfx | Probable RNA 2-phosphotransferase | 11 | 4.749 | 0.4318 |
| 271 | 1ug3 | Eukaryotic translation initiation factor 4 gamma 1 | 8 | 3.449 | 0.4312 |
| 272 | 3cv1 | Malate synthase A | 8 | 3.44 | 0.43 |
| 273 | 2da0 | Arf-GAP with SH3 domain, ANK repeat and PH domain-containing protein 1 | 9 | 3.869 | 0.4298 |
| 274 | 3gyr | Phenoxazinone synthase | 8 | 3.434 | 0.4292 |
| 275 | 3blv | Isocitrate dehydrogenase [NAD] subunit 1, mitochondrial | 11 | 4.716 | 0.4287 |
| 276 | 3cz8 | Putative sporulation-specific glycosylase ydhD | 7 | 2.991 | 0.4273 |
| 277 | 1eu1 | Dimethyl sulfoxide reductase | 9 | 3.844 | 0.4271 |
| 278 | 2c4k | Phosphoribosyl pyrophosphate synthetase-associated protein 1 | 7 | 2.989 | 0.4271 |
| 279 | 2ge9 | Tyrosine-protein kinase BTK | 7 | 2.984 | 0.4263 |
| 280 | 1v75 | Hemoglobin D subunit alpha | 8 | 3.407 | 0.4258 |
| 281 | 1g71 | DNA primase small subunit | 7 | 2.978 | 0.4254 |
| 282 | 1or7 | RNA polymerase sigma-E factor | 7 | 2.976 | 0.4252 |
| 283 | 1mqv | Cytochrome c | 7 | 2.975 | 0.425 |
| 284 | 1e6v | Methyl-coenzyme M reductase I subunit alpha | 9 | 3.824 | 0.4249 |
| 285 | 2c62 | Activated RNA polymerase II transcriptional coactivator p15 | 7 | 2.974 | 0.4249 |
| 286 | 1y8o | [Pyruvate dehydrogenase [lipoamide]] kinase isozyme 3, mitochondrial | 7 | 2.971 | 0.4244 |
| 287 | 3bpt | 3-hydroxyisobutyryl-CoA hydrolase, mitochondrial | 13 | 5.515 | 0.4242 |
| 288 | 1lnm | Bilin-binding protein | 7 | 2.966 | 0.4238 |
| 289 | 2qc7 | Endoplasmic reticulum protein ERp29 | 7 | 2.966 | 0.4238 |
| 290 | 2ysw | 3-dehydroquinate dehydratase | 8 | 3.388 | 0.4235 |
| 291 | 1a4e | Peroxisomal catalase A | 7 | 2.963 | 0.4232 |
| 292 | 1mg5 | Alcohol dehydrogenase | 8 | 3.384 | 0.423 |
| 293 | 1ll0 | Glycogenin-1 | 7 | 2.96 | 0.4229 |
| 294 | 1b6t | Phosphopantetheine adenylyltransferase | 7 | 2.96 | 0.4229 |
| 295 | 2nr0 | tRNA pseudouridine synthase A | 7 | 2.959 | 0.4228 |
| 296 | 3c5k | Histone deacetylase 6 | 7 | 2.958 | 0.4226 |
| 297 | 1u3r | Estrogen receptor beta | 7 | 2.957 | 0.4225 |
| 298 | 1wgw | Signal recognition particle 54 kDa protein | 7 | 2.955 | 0.4221 |
| 299 | 1tw2 | Carminomycin 4-O-methyltransferase | 7 | 2.954 | 0.4221 |

**Table S6.** *E. coli* protein targets for Aspergillipeptide A (10) and Emericellamide C (14) extracted from PharmMapper server and ranked by normalized fit score in descending order.

|  | **PDB ID** | **Protein name** | **Normalized fit score** |
| --- | --- | --- | --- |
| Aspergillipeptide A (10) | 1A5T | DNA polymerase III subunit delta | 0.7342 |
|  | 1gk9 | Penicillin G acylase | 0.7074 |
|  | 1U94 | Protein recA | 0.6382 |
|  | 2NR0 | tRNA pseudouridine synthase A | 0.6324 |
|  | 1M5Y | Chaperone surA | 0.6113 |
|  | 3GRH | Putative acyl-CoA thioester hydrolase ybhC | 0.5811 |
|  | 1NJF | DNA polymerase III subunit tau | 0.5692 |
|  | 1TRB | Thioredoxin reductase | 0.5573 |
|  | 1RKS | Ribokinase | 0.5439 |
|  | 2AY0 | Thioredoxin reductase | 0.5573 |
|  | 1XGE | Dihydroorotase | 0.4968 |
|  | 1OR7 | RNA polymerase sigma-E factor | 0.4937 |
|  | 1TXK | Glucans biosynthesis protein G | 0.4928 |
|  | 2D3W | Probable ATP-dependent transporter sufC | 0.491 |
|  | 1D8W | L-rhamnose isomerase | 0.4888 |
|  | 1DOS | Fructose-bisphosphate aldolase class 2 | 0.4851 |
|  | 1QSA | Soluble lytic murein transglycosylase precursor | 0.4809 |
|  | 1FDY | N-acetylneuraminate lyase | 0.4778 |
|  | 1JW2 | Hemolysin expression-modulating protein | 0.45 |
|  | 2HH8 | Uncharacterized protein ydfO | 0.415 |
|  | 1XPO | Transcription termination factor rho | 0.4141 |
|  | 2HI7 | Thiol:disulfide interchange protein dsbA | 0.4119 |
|  | 1WE5 | Alpha-xylosidase | 0.4087 |
|  | 1TVM | Galactitol-specific phosphotransferase enzyme IIB component | 0.4077 |
|  | 2EX8 | D-alanyl-D-alanine carboxypeptidase dacB | 0.4065 |
|  | 2VV5 | Small-conductance mechanosensitive channel | 0.4032 |
|  | 1D5Y | Right origin-binding protein | 0.3994 |
|  | 2NRO | Molybdopterin biosynthesis protein moeA | 0.3956 |
| Emericellamide C (14) | 1U94 | Protein recA | 0.7274 |
|  | 1M41 | Alkanesulfonate monooxygenase | 0.6743 |
|  | 1TJL | DnaK suppressor protein | 0.585 |
|  | 2BZ0 | GTP cyclohydrolase-2 | 0.5277 |
|  | 1NJK | Long-chain acyl-CoA thioesterase tesC | 0.4987 |
|  | 1IGW | Isocitrate lyase | 0.4921 |
|  | 1XPO | Transcription termination factor rho | 0.4875 |
|  | 1QSA | Soluble lytic murein transglycosylase precursor | 0.4867 |
|  | 2GB5 | NADH pyrophosphatase | 0.4843 |
|  | 2FIQ | D-tagatose-1,6-bisphosphate aldolase subunit gatZ | 0.4838 |
|  | 1XGE | Dihydroorotase | 0.4757 |
|  | 1S0W | Beta-lactamase TEM | 0.4754 |
|  | 1D8W | L-rhamnose isomerase | 0.472 |
|  | 2HZV | Nickel-responsive regulator | 0.4716 |
|  | 1OYZ | Protein yibA | 0.4713 |
|  | 2D3W | Probable ATP-dependent transporter sufC | 0.4711 |
|  | 1GUP | Galactose-1-phosphate uridylyltransferase | 0.4627 |
|  | 1QGD | Transketolase 1 | 0.4618 |
|  | 1NJF | DNA polymerase III subunit tau | 0.458 |
|  | 2VDE | Outer membrane protein tolC | 0.4495 |
|  | 1TXK | Glucans biosynthesis protein G | 0.4463 |
|  | 2EX8 | D-alanyl-D-alanine carboxypeptidase dacB | 0.4437 |
|  | 3GRH | Putative acyl-CoA thioester hydrolase ybhC | 0.4375 |
|  | 3CUO | Probable HTH-type transcriptional regulator ygaV | 0.4374 |
|  | 3CV1 | Malate synthase A | 0.43 |
|  | 1OR7 | RNA polymerase sigma-E factor | 0.4252 |
|  | 1B6T | Phosphopantetheine adenylyltransferase | 0.4229 |

**PDB: Protein Data Bank**

**Table S7.** Results of top 10 nodes calculated by 12 different centrality measures for PPI networks of Aspergillipeptide A (**10**) and Emericellamide C (**14**).

| **Aspergillipeptide A (10)** | | **Emericellamide C (14)** | |
| --- | --- | --- | --- |
| **1. Betweeness** | | | |
| dsbA | 663.8222474 | recA | 917.8076287 |
| trxB | 560.579681 | galK | 578.8595829 |
| recA | 443.465132 | ribD | 573.6844465 |
| sufS | 416 | polA | 553.2444771 |
| mobA | 390.829854 | tolC | 418.3844184 |
| holE | 389.1536764 | holE | 352.4857436 |
| trxA | 312.8951553 | macB | 351.8079249 |
| dnaE | 269.3616508 | coaE | 345.5556271 |
| bamD | 238.8812981 | rpe | 252.0313853 |
| polA | 227.405138 | rhaB | 177.1404171 |
| **2. BottleNeck** | | | |
| dsbA | 14 | recA | 28 |
| recA | 8 | polA | 12 |
| dnaX | 7 | ribD | 9 |
| trxB | 7 | tolC | 9 |
| bamD | 6 | holA | 6 |
| holE | 6 | galK | 6 |
| mobA | 6 | macB | 6 |
| truA | 6 | rpoE | 5 |
| polA | 5 | nusB | 5 |
| sufS | 5 | holE | 4 |
| **3. Closeness** | | | |
| dnaE | 33.16666667 | recA | 40.75 |
| recA | 32.95 | polA | 39.25 |
| polA | 31.5 | dnaX | 36.75 |
| dnaX | 31.2 | dnaE | 36.58333333 |
| dnaN | 30.7 | ribD | 36.08333333 |
| dsbA | 30.66666667 | dnaQ | 35.08333333 |
| holA | 30.33333333 | umuC | 34.25 |
| holB | 30.2 | dnaN | 34.25 |
| dnaQ | 30.2 | holB | 33.75 |
| holD | 29.66666667 | umuD | 33.75 |
| **4. Clustering Coefficient** | | | |
| ahpC | 1 | aceA | 1 |
| sufA | 1 | glcB | 1 |
| sufD | 1 | sufA | 1 |
| sufC | 1 | sufE | 1 |
| sufB | 1 | sufS | 1 |
| moaB | 1 | coaD | 1 |
| mog | 1 | sufD | 1 |
| moeA | 1 | sufC | 1 |
| moaE | 1 | rhaA | 1 |
| rhaA | 1 | sufB | 1 |
| **5. Degree** | | | |
| recA | 18 | recA | 23 |
| dnaE | 18 | polA | 21 |
| polA | 15 | dnaE | 19 |
| dnaX | 15 | dnaX | 18 |
| dnaN | 15 | umuC | 16 |
| holB | 14 | ribD | 16 |
| dnaQ | 14 | dnaQ | 16 |
| umuC | 13 | dnaN | 16 |
| holE | 13 | holB | 15 |
| holA | 13 | umuD | 15 |
| **6. DMNC** | | | |
| dnaQ | 0.822063185 | holE | 0.857962795 |
| umuD | 0.819558405 | holC | 0.831435407 |
| umuC | 0.817478379 | dinB | 0.817478379 |
| holB | 0.788279767 | holB | 0.791173898 |
| dnaN | 0.771144179 | umuC | 0.780755913 |
| lexA | 0.758089306 | dnaN | 0.780755913 |
| recQ | 0.738247057 | lexA | 0.780531199 |
| dnaX | 0.721069882 | holD | 0.778152303 |
| bamB | 0.713237441 | umuD | 0.771144179 |
| bamD | 0.692126905 | dnaQ | 0.762807501 |
| **7. EcCentricity** | | | |
| polA | 0.226190476 | dinB | 0.1875 |
| holD | 0.226190476 | dnaE | 0.1875 |
| trxB | 0.226190476 | umuC | 0.1875 |
| holA | 0.226190476 | holD | 0.1875 |
| bamA | 0.226190476 | holB | 0.1875 |
| dsbB | 0.226190476 | ribD | 0.1875 |
| holC | 0.226190476 | dinI | 0.1875 |
| dnaE | 0.226190476 | recQ | 0.1875 |
| dsbA | 0.226190476 | dnaX | 0.1875 |
| skp | 0.226190476 | rho | 0.1875 |
| **8. EPC** | | | |
| dnaE | 24.503 | recA | 28.604 |
| recA | 24.047 | polA | 28.517 |
| dnaN | 23.728 | dnaE | 28.476 |
| polA | 23.586 | dnaX | 28.396 |
| dnaX | 23.556 | dnaQ | 27.77 |
| dnaQ | 23.463 | dnaN | 27.761 |
| holB | 23.399 | holB | 27.685 |
| umuC | 23.386 | umuC | 27.672 |
| holC | 23.061 | umuD | 27.58 |
| holA | 23.008 | recQ | 26.557 |
| **9. MCC** | | | |
| dnaN | 497640 | dnaN | 895080 |
| dnaQ | 497520 | dnaQ | 894966 |
| dnaX | 491886 | umuC | 864720 |
| holB | 491880 | dnaE | 857666 |
| dnaE | 489736 | dnaX | 855496 |
| umuC | 455040 | holB | 849720 |
| umuD | 448560 | umuD | 817200 |
| holA | 443524 | polA | 500432 |
| holC | 404644 | recA | 462440 |
| holE | 403205 | dinB | 460800 |
| **10. MNC** | | | |
| recA | 17 | recA | 21 |
| dnaE | 16 | dnaE | 19 |
| dnaX | 15 | polA | 19 |
| dnaN | 15 | dnaX | 18 |
| holB | 14 | umuC | 16 |
| dnaQ | 14 | dnaQ | 16 |
| umuC | 13 | dnaN | 16 |
| polA | 13 | holB | 15 |
| holA | 13 | umuD | 15 |
| holC | 13 | ribD | 14 |
| **11. Radiality** | | | |
| dsbA | 5.444727891 | recA | 4.72983871 |
| recA | 5.412414966 | polA | 4.669354839 |
| dnaE | 5.396258503 | dnaX | 4.572580645 |
| polA | 5.347789116 | ribD | 4.560483871 |
| trxB | 5.331632653 | dnaE | 4.524193548 |
| dnaX | 5.31547619 | dnaQ | 4.5 |
| holA | 5.31547619 | umuC | 4.427419355 |
| skp | 5.267006803 | rho | 4.427419355 |
| holD | 5.25085034 | dnaN | 4.427419355 |
| trxA | 5.25085034 | holB | 4.415322581 |
| **12. Stress** | | | |
| dsbA | 2014 | recA | 2968 |
| holE | 1674 | polA | 2264 |
| trxB | 1532 | ribD | 1782 |
| recA | 1518 | holE | 1574 |
| dnaE | 1134 | galK | 1414 |
| sufS | 1024 | coaE | 1292 |
| trxA | 994 | tolC | 1056 |
| mobA | 924 | dnaX | 970 |
| rhaB | 920 | macB | 864 |
| polA | 910 | rpe | 852 |


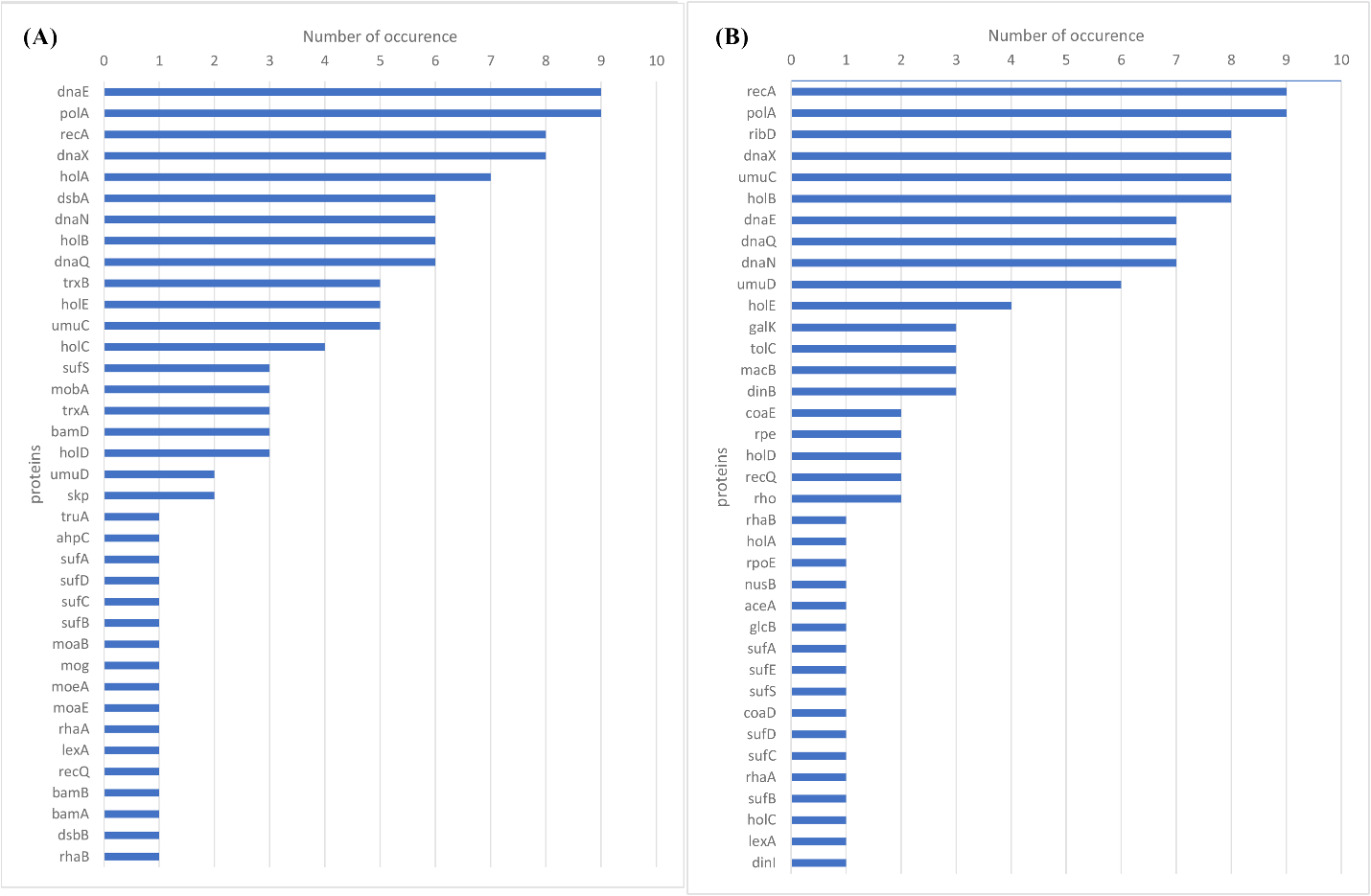


**Figure S1.** Number of occurrences for coinciding nodes from PPI networks. (A) Coinciding nodes of PPI network related to Aspergillipeptide A (**10**). (B) Coinciding nodes of PPI network related to Emericellamide C (**14**).
